# NOTCH1 is a mechanosensor in adult arteries

**DOI:** 10.1038/s41467-017-01741-8

**Published:** 2017-11-20

**Authors:** Julia J. Mack, Thiago S. Mosqueiro, Brian J. Archer, William M. Jones, Hannah Sunshine, Guido C. Faas, Anais Briot, Raquel L. Aragón, Trent Su, Milagros C. Romay, Austin I. McDonald, Cheng-Hsiang Kuo, Carlos O. Lizama, Timothy F. Lane, Ann C. Zovein, Yun Fang, Elizabeth J. Tarling, Thomas Q. de Aguiar Vallim, Mohamad Navab, Alan M. Fogelman, Louis S. Bouchard, M. Luisa Iruela-Arispe

**Affiliations:** 1 Department of Molecular, Cell & Developmental Biology, University of California, Los Angeles, CA 90095 USA; 2 Institute for Quantitative and Computational Biology, University of California, Los Angeles, CA 90095 USA; 3 Department of Bioengineering, University of California, Los Angeles, CA 90095 USA; 4 Interdepartmental Graduate Program in Molecular, Cellular and Integrative Physiology, University of California, Los Angeles, CA 90095 USA; 5 Department of Neurology, David Geffen School of Medicine, University of California, Los Angeles, CA 90095 USA; 6 Molecular Biology Interdisciplinary Graduate Program, Molecular Biology Institute, University of California, Los Angeles, CA 90095 USA; 7 Department of Biological Chemistry, University of California, Los Angeles, CA 90095 USA; 8 Department of Medicine, University of Chicago, Chicago, IL 60637 USA; 9 Cardiovascular Research Institute, University of California, San Francisco, CA 94158 USA; 10 Department of Ob-Gyn, David Geffen School of Medicine, University of California, Los Angeles, CA 90095 USA; 11 Molecular Biology Institute, University of California, Los Angeles, CA 90095 USA; 12 Department of Medicine, David Geffen School of Medicine, University of California, Los Angeles, CA 90095 USA; 13 Department of Chemistry and Biochemistry, University of California, Los Angeles, CA 90095 USA

## Abstract

Endothelial cells transduce mechanical forces from blood flow into intracellular signals required for vascular homeostasis. Here we show that endothelial NOTCH1 is responsive to shear stress, and is necessary for the maintenance of junctional integrity, cell elongation, and suppression of proliferation, phenotypes induced by laminar shear stress. NOTCH1 receptor localizes downstream of flow and canonical NOTCH signaling scales with the magnitude of fluid shear stress. Reduction of NOTCH1 destabilizes cellular junctions and triggers endothelial proliferation. NOTCH1 suppression results in changes in expression of genes involved in the regulation of intracellular calcium and proliferation, and preventing the increase of calcium signaling rescues the cell–cell junctional defects. Furthermore, loss of *Notch1* in adult endothelium increases hypercholesterolemia-induced atherosclerosis in the descending aorta. We propose that NOTCH1 is atheroprotective and acts as a mechanosensor in adult arteries, where it integrates responses to laminar shear stress and regulates junctional integrity through modulation of calcium signaling.

## Abstract (web summary)

The arterial wall is subjected to mechanical forces that modulate endothelial cell responses. Here, Mack and colleagues identify a novel role for Notch1 as a mechanosensor in adult arteries, where it ensures junctional integrity through modulation of calcium signalling and limits atherosclerosis.

## Introduction

The vascular wall is subjected to physical forces resulting from the rhythmic activities of the heart. As the innermost lining of all blood vessels, the endothelium is uniquely responsive to these forces, particularly shear stress, which is transduced by endothelial cells into molecular signals that coordinate homeostatic responses^1–4^. Laminar shear stress induces elongation of endothelial cells^5,6^, suppression of endothelial cell proliferation, redistribution of focal adhesions, reassembly of junctional complexes, and cytoskeletal organization^7,8^. These cellular responses are complex and require both shear stress sensors and a robust cohort of effector molecules that coordinate rapid changes and physiological adaptations.

Importantly, variations in blood flow result in altered hemodynamic forces throughout the vasculature^9^. These hemodynamic forces play an important role in regulating the phenotype and gene expression of endothelial cells in different regions of the arterial wall^10–13^. The descending thoracic aorta is defined by high laminar shear stress and its resulting endothelial gene profile is “atheroprotective”^14^. In contrast, the inner curvature of the aortic arch is characterized by disturbed blood flow with oscillatory shear stress that promotes an “atheroprone” expression profile^15–17^. In this manner, atherosclerosis is known to occur largely in arterial regions exposed to oscillatory shear stress^17^. Because of the clinical impact of these responses, the mechanisms of endothelial mechanotransduction are of great interest.

Mechanosensors act as the initial responders to changes in the mechanical environment^18,19^. Several of these have been identified including integrins, ion channels, G-protein-coupled receptors, and endothelial cell–cell junctional proteins^20^. However, the picture of the key contributors involved in flow mechanosensing remains incomplete. Recently, NOTCH1 has been shown to be flow-responsive and involved in modulating the expression of endothelial inflammatory genes^21–23^. Considering that NOTCH1 expression is retained in adult arteries^21^ and activation of this receptor is dependent on physical forces^24^, we investigated the flow-responsive nature of NOTCH1 signaling to determine its biological significance in adult arteries. Our findings indicate that NOTCH1 signaling responds to laminar flow and that this response scales with the magnitude of shear stress. Furthermore, we show that NOTCH1 protein is able to sense laminar flow by rapidly locating to the downstream pole relative to the flow direction. Our results further reveal that NOTCH1 is required to maintain junctional integrity, promote cell elongation in response to flow, and prevent atherosclerosis in the context of hypercholesterolemia. Overall, these findings indicate that NOTCH1 signaling is required in adult arteries to interpret hemodynamic forces and initiate appropriate biological responses required for vascular homeostasis and atheroprotection.

## Results

### NOTCH1 signaling is increased by shear stress

**Fig. 1:**
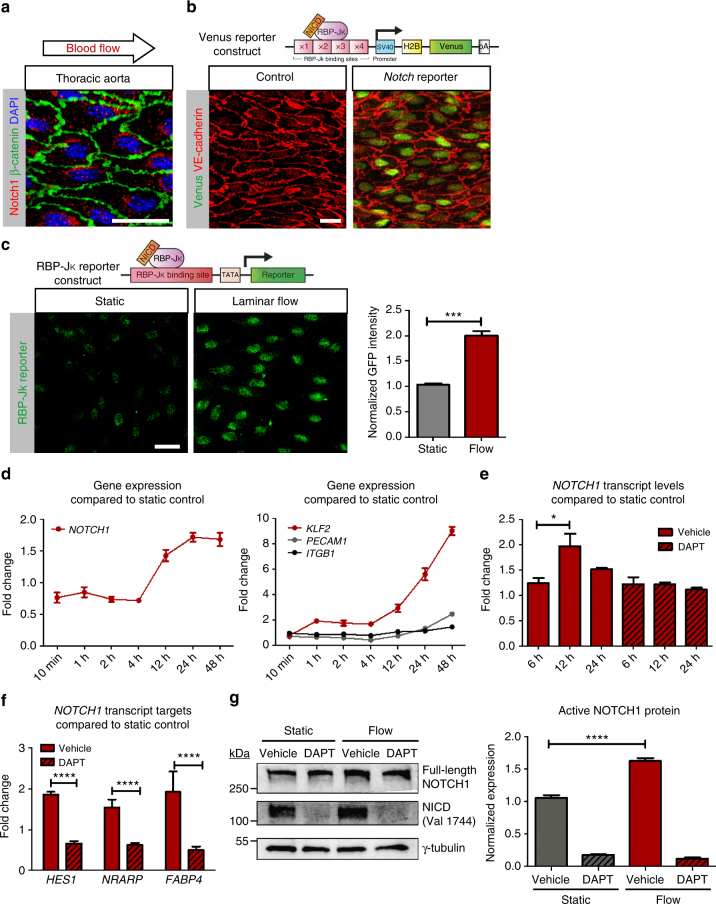
Notch1 is activated by shear stress in vitro. **a** En face confocal imaging of wildtype (C57BL/6) adult mouse thoracic endothelium shows Notch1 (red). Staining was done in 20 mice of different strains with identical results, scale bar = 20 µm. **b** En face imaging of Venus Notch reporter mouse (RBP-Jk:H2B-Venus transgenic) compared to control aorta imaged using identical settings. Note that levels of reporter vary amongst cells indicating distinct degrees of activation in the intima at a given time. Scale bar = 20 µm. **c** HAECs transfected with GFP-RBP-Jk reporter showed a two-fold increase in GFP signal intensity under flow (20 dynes cm^−2^) compared to static control at 24 h (plotted is average GFP intensity ± SEM of ~120 cells per condition, from three biological replicates). *T-*test, ***P < 0.001. Scale bar = 20 µm. **d** Onset of flow (from 10 min to 48 h) increases *NOTCH1* mRNA after 4 h for HAECs. Transcript levels of *KLF2*, *PECAM1*, and *ITGB1* were also profiled over time and compared to static controls. Data shown include mean ± SEM, *n* = 4 biological replicates. **e** *NOTCH1* transcript levels assessed by NanoString chip technology. HAECs were subjected to laminar shear stress for 6, 12, and 24 h with γ-secretase inhibitor DAPT or vehicle and compared to vehicle treated static controls. Graph bars represent mean ± SEM, *n* = 4 biological replicates, statistical significance for 6 h and 12 h: Wilcoxon rank-sum test, *P* = 0.0236. **f** Flow response of NOTCH1-target genes *HES1*, *NRARP*, and *FABP4* was determined in HAECs after 12 h of laminar flow while treated with DAPT or vehicle and compared to vehicle-treated static controls. Graph bars represent mean ± SEM, *n* = 5 biological replicates. *T-*test, ***P < 0.001. **g** Endothelial monolayers were flow-conditioned for 24 h in the presence of DAPT or vehicle and protein lysates were analyzed by immunoblot to determine NICD expression levels (*n* = 5 biological replicates, ****P < 0.0001 by *t-*test)

Notch signaling is necessary for arterial specification during development^25–28^. Importantly, immunohistochemistry of mouse aorta revealed that Notch1 protein was abundant in endothelial cells (Fig. 1a) indicating its continuous expression in adult arteries. Additionally, Notch1 activity was robust, as assessed by reporter mice (RBP-Jk:H2B-Venus strain^29^). Venus reporter protein was observed in the endothelium of the descending aorta (Fig. 1b) and the carotid artery (Supplementary Fig. 1a), indicating that Notch1 signaling was active in quiescent, non-angiogenic, aortic endothelium.

To determine the effect of shear stress on *NOTCH1* transcripts, we transfected human aortic endothelial cells (HAECs) with GFP-RBP-Jk reporter constructs and subjected them to static and flow conditions. Fluorescence imaging revealed a two-fold increase in GFP signal in cultures under laminar flow compared to static conditions (Fig. 1c); this correlated with nuclear presence of NOTCH1 protein (Supplementary Fig. 1b). Transcripts were also increased by 12 h after exposure to flow (Fig. 1d). By 24 h, transcript levels were 1.6-fold higher than static controls, an increase comparable to PECAM1, a known component of a mechanosensory complex^30^, which is activated two-fold at 48 h under flow. *KLF2* mRNA was used as a positive control to confirm flow conditions (Fig. 1d, right plot). Next, HAECs exposed to flow were subjected to γ-secretase inhibitor DAPT or vehicle for 6, 12 and 24 h. NOTCH1 expression was increased by nearly two-fold after 12 h of flow. Importantly this rise in transcript levels was blocked by DAPT (Fig. 1e) suggesting the need for a positive feedback loop mediated by NOTCH1 signaling. We then assessed transcripts for NOTCH1 targets at 12 h of flow compared to static controls to determine whether flow-dependent *NOTCH1* gene activation translated into activation of NOTCH downstream effectors. NOTCH1 targets *HES1*, *NRARP*, and *FABP4* were increased by flow and blocked by DAPT (Fig. 1f). To determine whether *NOTCH1* transcriptional activation correlated with protein levels, we evaluated endothelial cells under static and flow conditions in the presence of DAPT or vehicle for 24 h. Active NOTCH1 protein (NICD) levels increased 1.7-fold under flow compared to static control (Fig. 1g and Supplementary Fig. 1c). This activation was blocked by DAPT treatment in both static and flow conditions. These in vitro experiments provide evidence that NOTCH1 is activated under laminar shear stress and that laminar shear stress results in positive feedback on NOTCH signaling.

### Nuclear NICD responds to stepwise increases in shear stress

**Fig. 2:**
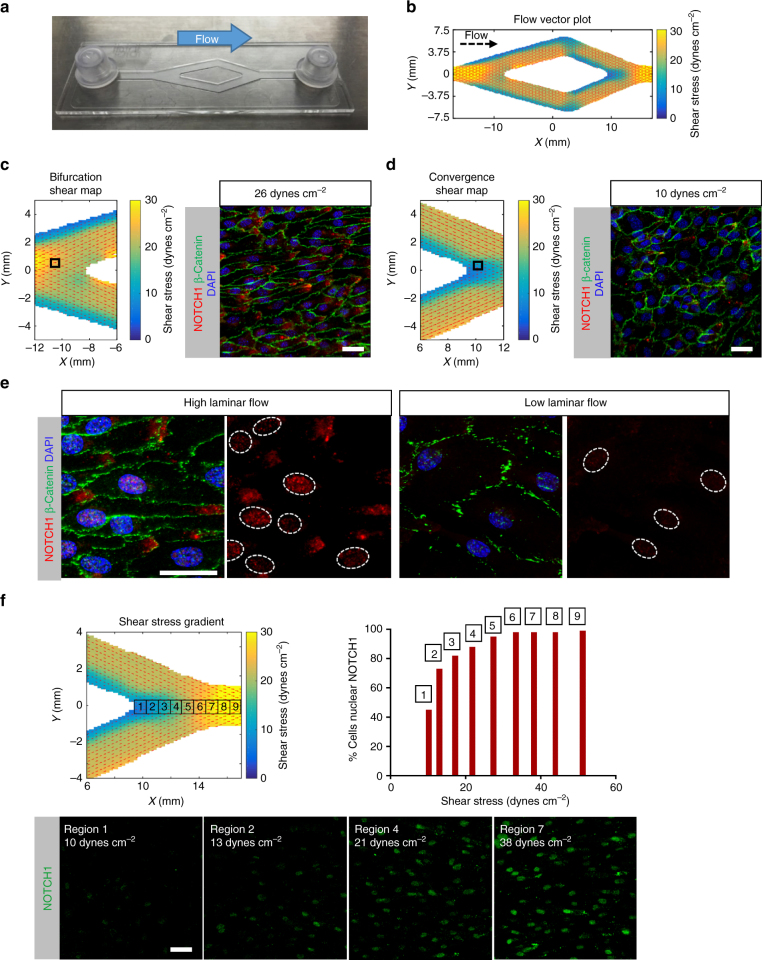
Shear stress potentiates activation and consequent nuclear translocation of NOTCH1 protein. **a** Endothelial cells (HAECs) were plated to confluency on a y-slide (ibidi) and subjected to laminar flow (9 mL min^−1^ applied). **b** MRI flow mapping of y-slide in the presence of flow-conditioned HAECs (48 h) generated flow vector plots across all regions of the y-slide (*n* = 5). **c** Flow vector plot of high flow speed (26 dynes cm^−2^) region and corresponding HAEC morphology. Notice cell shape defined by β-catenin expression at cell–cell borders (green) and polarized NOTCH1 (red) protein. Scale bar = 20 µm. **d** Flow vector plot of low shear stress (10 dynes cm^−2^) region and corresponding HAEC morphology with β-catenin (green) and NOTCH1 (red). Scale bar = 20 µm. **e** Staining for NOTCH1 (red) in high-flow region compared to low-flow region. Dashed white ovals reveal increase of nuclear NOTCH1 protein under high flow compared to low flow. Scale bar = 20 µm. **f** Flow vector plot of the back end of the y-slide for nine regions of interest. NOTCH1 (green) nuclear staining was quantified for each of the nine regions and plotted as a function of the measured wall shear stress. For each measurement, ~100 cells were evaluated using *n* = 5 biological replicates, scale bar = 20 µm

Using a Y-shaped fluidic chamber (Fig. 2a), we generated variable flow speeds on a single monolayer of HAECs. Magnetic resonance imaging (MRI) flow mapping^31^ was used to obtain a vector field plot of flow velocities in the presence of a flow-conditioned monolayer. From the magnitude of the MRI-derived flow speed (m s^−1^), fluid shear (dynes cm^−2^) values were calculated using an assumption of flow between infinitely wide parallel plates and overlaid on the flow vector field plot as a colormap (Fig. 2b). Focusing on a region of high shear (26 dynes cm^−2^), endothelial cells were elongated and aligned in the flow direction (Fig. 2c) as assessed by staining for β-catenin. Staining for NOTCH1 revealed polarization of the protein as clusters at the downstream pole of the cell (Fig. 2c). In contrast, regions of low shear (10 dynes cm^−2^) showed lack of cell elongation (Fig. 2d). Side-by-side comparisons of high and low shear stress regions revealed a marked increase of NOTCH1 protein in the nuclei of endothelial cells under high laminar flow (Fig. 2e). To determine whether nuclear NOTCH1 levels were responsive to shear stress levels, we measured nine regions of interest (ROIs). We then set a threshold value for NOTCH1 protein and determined the percentage of cells with nuclear NOTCH1 in each ROI (Fig. 2f) uncovering a positive correlation of nuclear translocation of NOTCH1 with increasing shear stress. The relationship was logarithmic, with large increases in NOTCH1 nuclear translocation at initially small increases in shear stress, followed by a response plateau starting at 26 dynes cm^−2^. The findings implied that NOTCH1 activation was finely tuned with flow and reached a maximal activation (saturation) above a specific flow-induced shear stress level.

### Laminar fluid flow polarizes NOTCH1 protein

**Fig. 3:**
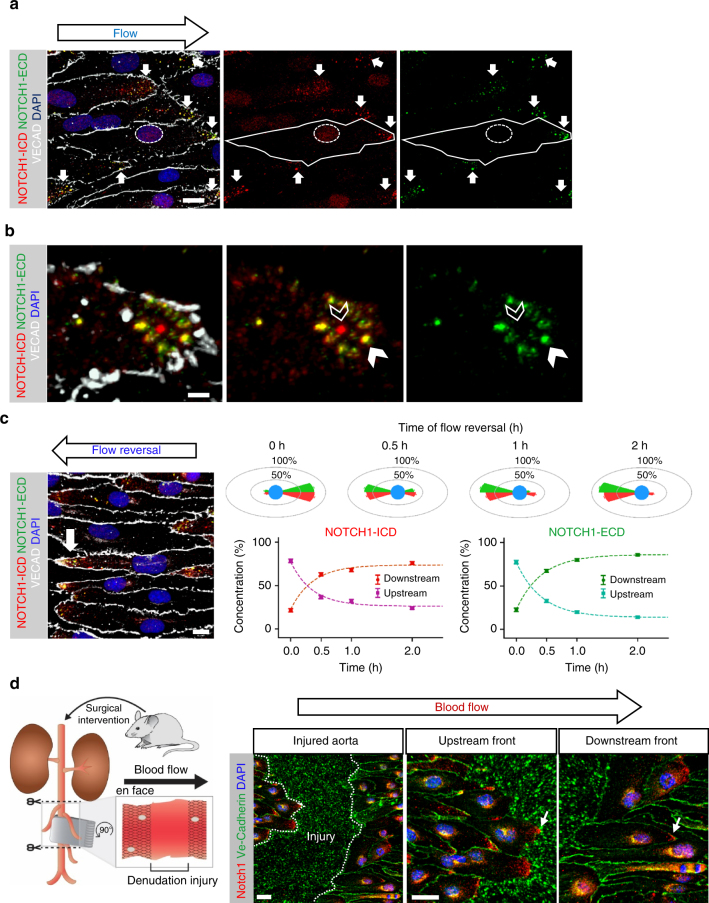
NOTCH1 is polarized downstream of flow. **a** Endothelial cells (HAECs) subjected to high laminar flow (20 dynes cm^−2^) display significant cellular elongation, revealed by VECAD (white) and distribution of NOTCH1 intracellular domain (ICD, red) and extracellular domain (ECD, green) at the downstream pole downstream pole, as marked by solid white arrows. Dashed line marks a cell nucleus confirming the nuclear presence of NOTCH1 ICD without ECD. Scale bar = 10 µm. **b** Downstream pole of an endothelial cell subjected to high laminar flow showing NOTCH1 ECD (green) and ICD (red). Co-localization of ECD and ICD is indicated by the solid arrowhead and intracellular activated NOTCH1 is marked by the open arrowhead; scale bar = 3 µm. **c** Upon flow-direction reversal, NOTCH1 ICD and ECD protein redistributes to the new downstream pole; scale bar = 10 µm. Kinetics of the protein redistribution was measured and the typical time for repolarization (dashed lines) was ~30 min (*n* ≥ 116 individual cells for each time point measured). **d** Arterial denudation injury of wildtype (C57BL/6) adult mouse to generate injury region (marked by dotted white line) with two migrating endothelial fronts. Note the presence of Notch1 (marked by white arrows) at the downstream pole of the cell regardless of migration direction (at least 40 cells at the wound margins were evaluated in three distinct wounded animals). Scale bars = 20 µm

To distinguish NOTCH1 protein polarization from NOTCH1 signaling, we used distinct antibodies for NOTCH1 intracellular domain (ICD) or extracellular domain (ECD), with the objective of visualizing both total protein and activated (cleaved) protein. Analysis of NOTCH1 protein distribution under flow revealed NOTCH1 localization to cluster at the downstream pole for both the ICD and ECD with some ICD observed in the nucleus (Fig. 3a). In these cells, the ECD was noted only downstream of flow direction. Higher magnification showed ECD at the downstream pole where we also found ICD (Fig. 3b, solid arrowhead). Although we observed the majority of polarized ICD and ECD co-localizing in yellow (Fig. 3b), clusters of cleaved NOTCH1 were also present (Fig. 3b, open arrowhead). These results suggested that both full-length and active NOTCH1 protein were polarized downstream of flow in aligned endothelial cells. To determine whether NOTCH1 protein polarization was flow direction dependent, we reversed the flow direction for 24 h and observed that NOTCH1 protein re-distributed to the new downstream pole (Fig. 3c). The kinetics of protein re-distribution was evaluated for over 100 individual cells at multiple time points to reveal that re-polarization occurred within 1 h of flow direction reversal (Supplementary Fig. 2). To confirm whether this flow-induced polarity was biologically relevant or merely an artifact of in vitro culture, we performed a surgical arterial denudation injury^32^ on the mouse thoracic aorta (Fig. 3d schematic) and waited 48 h post operation to allow for progression of the two migrating endothelial fronts of the wound. Staining for Notch1 protein showed localization downstream of blood flow direction in both migrating fronts, indicating that Notch1 protein polarizes downstream of flow regardless of cell migration direction (Fig. 3d).

### NOTCH1 is required for endothelial cell responses to flow

**Fig. 4:**
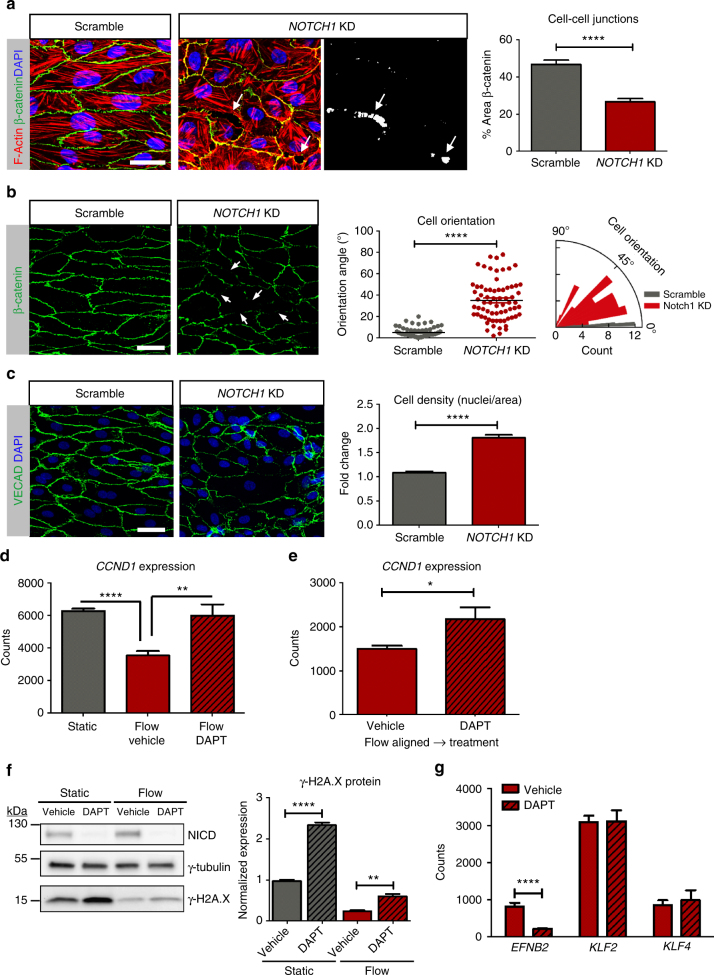
NOTCH1 is required for maintenance of endothelial cell–cell junctions, cell alignment, and suppression of proliferation under flow. Endothelial cells (HAECs) were treated with siRNA for *NOTCH1* or Scramble and flow-conditioned for 48 h (20 dynes cm^−2^). **a** F-Actin and β-catenin revealed gaps between cells as indicated with arrows and in white on inverted image; scale bar = 20 µm. Cell–cell junctions were quantified by percent area β-catenin coverage, shown is mean ± SEM from five distinct cultures obtained from five biological replicates. *T-*test, ****P < 0.0001. **b** β-Catenin staining to reveal cell shape and determine cell orientation angle (positive value of degrees) with respect to the flow direction. At least 70 cells were evaluated per condition across three biological replicates; scale bar = 20 µm. *T-*test ****P < 0.0001. **c** Increased cell density for *NOTCH1* KD compared to control by quantification of nuclei counts; scale bar = 20 µm. Graphs show data obtained from four biological replicates, >100 cells per replicate, and expressed as mean ± SEM. *T-*test ****P < 0.0001. **d** *CCND1* transcript was analyzed in HAECs cultured statically or flow-treated with 50 µM DAPT or volume equivalent DMSO for 24 h by NanoString nCounter Gene Expression. Graph bars represent mean ± SEM, *n* = 5. *T-*test **P < 0.01, ****P < 0.0001. **e** *CCND1* transcript is increased after blocking NOTCH1 signaling for HAEC monolayers that were flow-aligned for 48 h and subsequently treated with DAPT or vehicle under flow for an additional 12 h. Graph bars represent mean ± SEM, *n* = 5. *T-*test *P < 0.05. **f** Monolayers were subjected to flow for 24 h with 50 µM DAPT or vehicle and protein lysates analyzed by immunoblot to quantify expression of γ-H2A.X to show a 2.7-fold increase in γ-H2A.X expression for DAPT treated cultures (*n* = 4, *T-*test **P < 0.01). **g** NanoString nCounter Gene Expression for *EFNB2*, *KLF2*, and *KLF4* transcripts for HAECs cultured under flow for 24 h in the presence of 50 µM DAPT or DMSO revealed reduction in transcripts for *EFNB2*, but no statistically significant change for *KLF2* or *KLF4*. Graph bars represent mean ± SEM, *n* = 5. *T-*test ****P < 0.0001

To determine the relevance of NOTCH1 under flow conditions, protein depletion by siRNA knockdown (KD) and Scramble (control) endothelial cells were evaluated under flow (Supplementary Fig. 3a). NOTCH1 protein and transcript levels were assessed over time to validate experimental time points (Supplementary Fig. 3b–d). NOTCH1 KD monolayers displayed gaps between endothelial cells (Fig. 4a) with reduction of flow-induced orientation compared to control monolayers (Fig. 4b). We observed a significant decrease in the percent area of β-catenin coverage, correlating with the presence of gaps (marked in white) (Fig. 4a). Furthermore, discontinuous cell–cell junctions, revealing gaps between cells, were noted in the DAPT treated cultures exposed to flow (Supplementary Fig. 4a, b). We also calculated the orientation angle (degrees) of HAECs with respect to the flow direction^33^. This revealed that while the control monolayers exhibited flow alignment, within 10° of flow direction, NOTCH1 KD cells were less aligned, averaging 37° off of the flow direction, with a much wider distribution of angles (Fig. 4b). To further inspect NOTCH1 KD endothelial cell response to flow, we stained for Golgi complex markers (Supplementary Fig. 4c), which have been shown to polarize either downstream or upstream of the nucleus in response to laminar flow in endothelial cells^34^. For control monolayers, the vast majority (>80%) of cells presented with the Golgi located either downstream or upstream of flow direction; however the NOTCH1 KD cells showed a lack of polarization with <30% of the cells showing Golgi polarization. Next, we determined the elongation factor of endothelial cells by staining for β-catenin and measuring the cell length along the flow direction divided by the cell width^5^. Control monolayers displayed an average elongation factor of 4.5 while the NOTCH1 KD monolayers had a reduced elongation factor of 2 (Supplementary Fig. 4d). Once flow aligned, NOTCH signaling was continually required to maintain elongated cell phenotype under flow (Supplementary Fig. 4e). Combined, these data indicate that NOTCH1 is required for endothelial cells to maintain cell–cell junctions, elongated cell morphology, and alignment with flow.

### Absence of NOTCH1 promotes endothelial cell proliferation

Evaluation of flow-treated NOTCH1 KD monolayers revealed a two-fold increase in cell density compared to control under flow (Fig. 4c). Cell density was also assessed in cultures subjected to flow and treated with DAPT or vehicle with identical findings (Supplementary Fig. 5a). Interestingly, assessment of *CCND1* (Cyclin D1) transcript levels showed suppression under flow exposed to vehicle, but levels were elevated and close to identical between the static and DAPT monolayers exposed to flow (Fig. 4d). We found that endothelial alignment and flow conditioning in the presence of NOTCH1 signaling were not sufficient to prevent the subsequent upregulation of *CCND1* once NOTCH1 signaling was inhibited (Fig. 4e). We confirmed the increase in Cyclin D1 protein levels in the absence of NOTCH1 in independent cultures by immunoblot (Supplementary Fig. 5b) and found that γ-H2A.X (phospho-S139), a marker of DNA double-strand breaks recruited during mitosis^35^, was increased by almost three-fold in flow-conditioned monolayers when NOTCH1 signaling was inhibited (Fig. 4f). Increased γ-H2A.X protein was also observed in static cultures for NOTCH1 KD cells compared to control (Supplementary Fig. 5c). Further transcriptional profiling of cells cultured under flow with and without NOTCH blockade, indicated that arterial marker EPHRIN B2 was significantly downregulated, but other flow-responsive genes, i.e., *KLF2* and *KLF4*, were unaffected (Fig. 4g). This implied that NOTCH1 signaling was required for maintenance of arterial phenotype and suppression of cell cycle, despite the normal response of other flow-responsive genes indicating that NOTCH1 is necessary for the suppression of cell proliferation.

### Deletion of *Notch1* in mice reproduced in vitro findings

**Fig. 5:**
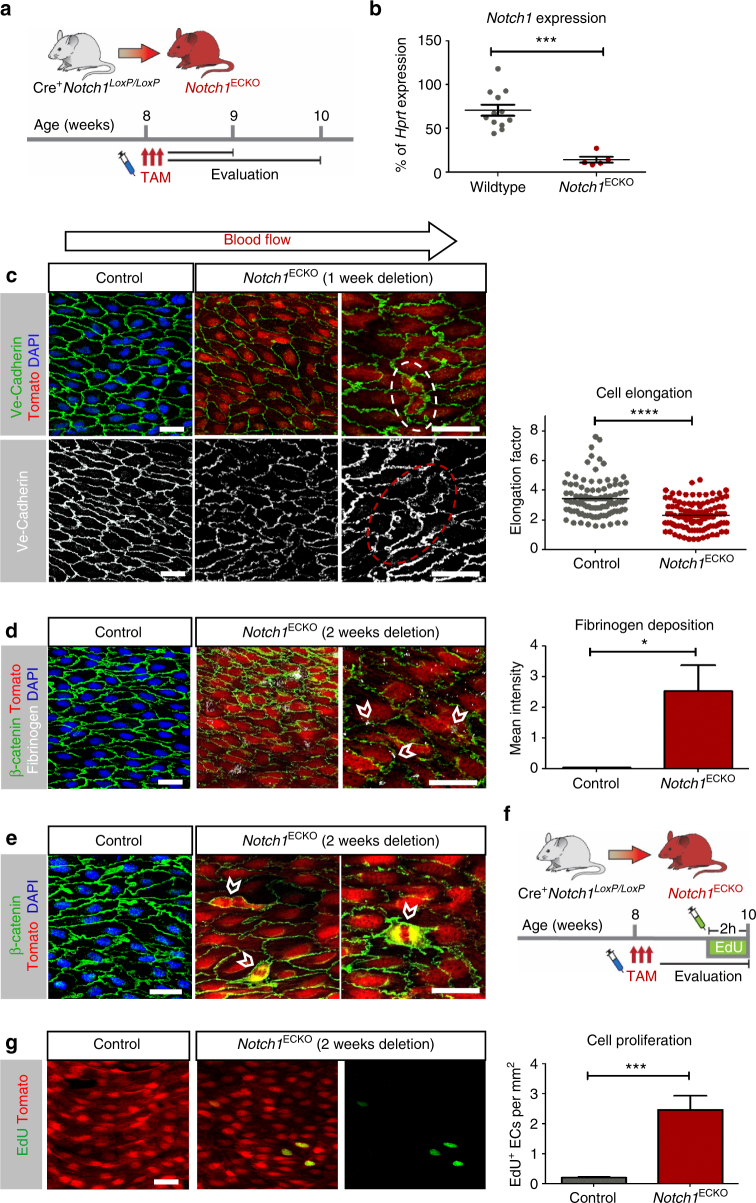
Deletion of *Notch1* results in regression of cell alignment, compromised cell–cell junctions, and enhanced proliferation. **a** Schematic to illustrate time line for in vivo model of inducible deletion of endothelial *Notch1* (*Notch1* ^ECKO^). Cre^+^ *Notch1* ^loxP/loxP^ mice after 8 weeks of age received three intraperitoneal injections of tamoxifen as did age-matched control mice at specified time points. **b** *Notch1* transcript levels from descending aortae of adult WT mice and *Notch1* ^ECKO^ mice 2 weeks post tamoxifen measured by qPCR analysis (*n* = 12 WT and *n* = 5 *Notch1* ^ECKO^). *T-*test ****P < 0.0001. **c** En face confocal imaging of the endothelium of the descending aorta for *Notch1* ^ECKO^, 1 week post deletion, and control mice (Cre^−^ *Notch1* ^loxP/loxP^); all mice were injected with tamoxifen. Ve-Cadherin revealed instances of endothelial cells oriented perpendicular to blood flow direction (highlighted by dashed ovals). Quantification of cell orientation, measured by elongation factor (cell length along flow direction divided by cell width) for *Notch1* ^ECKO^ vs. control (Cre^−^ *Notch1* ^loxP/loxP^). All mice were injected with tamoxifen and evaluated 1 week later (100 cells were measured from 5 mice for each genotype, *T-*test ****P < 0.0001). **d** En face imaging of descending aortas 2 weeks post tamoxifen revealed fibrinogen deposited between endothelial cells (open arrow heads) in *Notch1* ^ECKO^ animals, but not in age-matched tamoxifen-injected controls. Determination of fibrinogen was obtained by mean fluorescence intensity in areas of 500 µm^2^ from five mice of each genotype. Graph bars represent mean ± SEM. *T-*test *P < 0.05. **e** Proliferating endothelial cells, as evidenced by the mitotic figures (solid arrow heads), in *Notch1* ^ECKO^ aortas but not in control (Cre^−^ *Notch* ^loxP/loxP^). **f** Experimental design to quantify endothelial proliferation by EdU incorporation in the descending aortae of control and *Notch1* ^ECKO^ animals at 2 weeks post tamoxifen. **g** Representative en face images and quantification of EdU-positive endothelial cells per unit area of aorta (EdU^+^ endothelial cells per mm^2^). *Notch1* ^ECKO^ and control (Cre^+^ tdTomato reporter). EdU incorporation is visualized in green. Graph bars represent mean of ~2 cm^2^ segments of the aorta evaluated from six mice per genotype ± SEM. *T-*test ***P < 0.001. Scale bars = 20 µm

To further explore biological relevance, we used a transgenic mouse model to enable endothelial-specific inducible deletion of *Notch1* in the adult (>8 weeks of age, *Notch1* ^ECKO^) (Fig. 5a). *Notch1* deletion was confirmed by qPCR (Fig. 5b) and activation of Cre-recombinase at the cellular level was validated by tdTomato reporter expression (Fig. 5c). Confocal imaging of the endothelium and staining for endothelial junctional marker Ve-cadherin revealed changes in endothelial cell orientation, as evidenced by instances of cells orientating perpendicular to flow direction at 1 week post *Notch1* deletion (Fig. 5c; dashed ovals). Measuring cell shape and elongation factor for age-matched control littermates vs. *Notch1* ^ECKO^ mice, we found a statistically significant reduction in endothelial cell elongation factor from 3.5 to 2.3. These findings indicate that *Notch1* signaling was required to maintain flow-induced polarity of endothelial cells. We also found discontinuity in cell–cell junctions in the *Notch1* ^ECKO^ endothelium, more clearly noted when aortas were stained for fibrinogen^36^ and quantified for fibrinogen deposition (Fig. 5d). At 2 weeks post endothelial *Notch1* deletion, we also observed proliferating endothelial cells (white arrow heads) in the descending aorta (Fig. 5e). Quantification of EdU incorporation for *Notch1* ^ECKO^ aortic endothelium compared to tamoxifen injected littermate controls (Fig. 5f) indicated a significant enhancement (over two-fold) in endothelial cell proliferation as shown by green fluorescence signal (Fig. 5g). These experiments point to an essential requirement for *Notch1* in flow-induced responses associated with cell–cell junctions, endothelial cell polarity, and suppression of proliferation in adult arteries.

### Transcriptional changes induced by NOTCH1 reduction

**Fig. 6:**
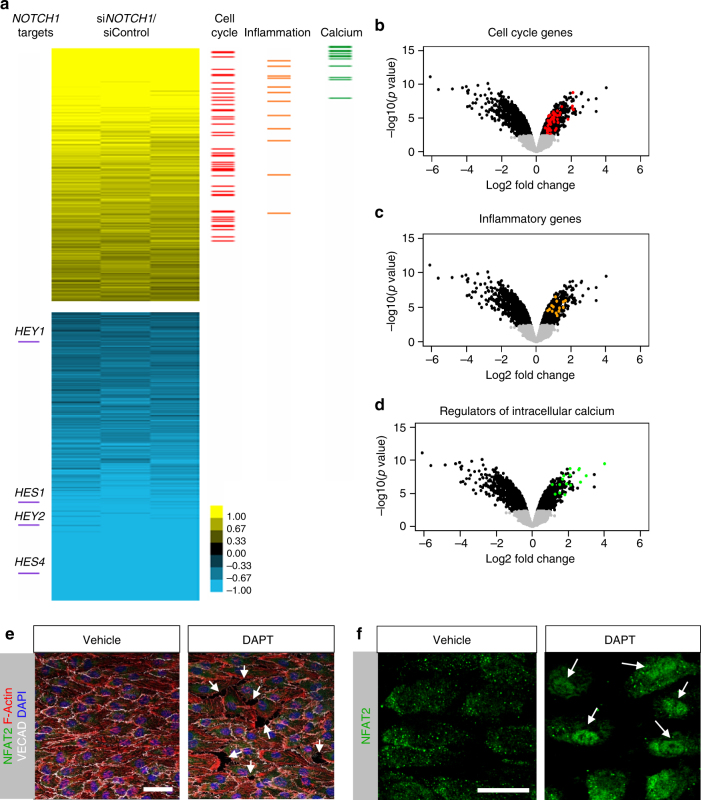
Transcriptome of siNOTCH1 indicates changes in cell cycle, inflammatory genes, and regulators of calcium. **a** Cultures of HAECs treated with siNOTCH1 and siControl were analyzed for transcriptional changes by microarray. Heatmap to display differentially expressed genes (≥0.5 (log2) upregulated or ≤ −0.5 (log2) downregulated with *P* < 0.001) marking NOTCH1 target genes and upregulated cell cycle, inflammatory genes and regulators of intracellular calcium. (**b**–**d**) Volcano plot visualization of the differentially expressed genes between siNOTCH1 and siControl. Colors indicate as follows: gray—no differential expression, black—genes whose fold change is either ≤0.50 or ≥1.5, red—genes known to contribute to the regulation of cell cycle, orange—genes involved in inflammation, and green—genes known to be regulators of intracellular calcium. (**e**, **f**) Confocal images of HAECs exposed to flow for 48 h in the presence of γ-secretase inhibitor DAPT or vehicle and stained for F-actin, VECAD, and NFAT2. Note the presence of nuclear NFAT2 and gaps between cells only in DAPT-treated monolayers (marked by white arrows). Scale bars = 20 µm

Transcriptome analysis of si*NOTCH1* and control HAECs were evaluated to identify differences^21^. A heatmap revealed that reduction of NOTCH1 affected cell cycle, inflammation, and calcium-dependent pathways (Fig. 6a). Volcano plots of the data visualizes the differentially expressed genes in black with specific cell cycle genes highlighted in red, inflammatory genes highlighted in orange, and genes regulated by intracellular calcium in green (Fig. 6b–d; Supplementary Tables 1–3 lists highlighted genes). NFATs are a family of calcium-dependent transcription factors that are stimulated by a rise in cytoplasmic Ca^2+^ and the translocation of NFAT proteins to the nucleus requires the phosphatase calcineurin^37^, which is activated by increase of cytosolic Ca^2+^. Staining for NFAT2 in endothelial monolayers under flow revealed gaps (Fig. 6e, arrows indicating gaps between cells) and nuclear NFAT2 (Fig. 6f, arrows) in monolayers where NOTCH1 signaling was blocked by γ-secretase inhibitor DAPT. These data pointed to a dysregulation of intracellular Ca^2+^ in endothelial cells lacking NOTCH1 signaling.

### Intracellular Ca^2+^ is modulated by NOTCH1 under flow

**Fig. 7:**
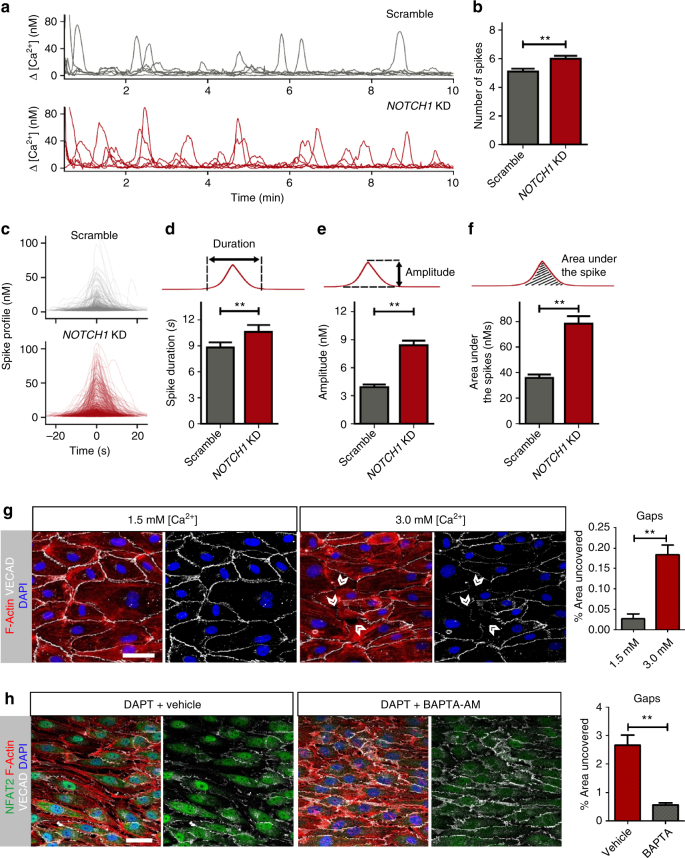
Reduction in NOTCH1 increases Ca^2+^ signaling under flow. **a** Typical examples of Ca^2+^ responses using OGB-1-loaded HAECs after onset of flow at *t* = 0 for Scramble (top) and NOTCH1 KD (bottom). **b**–**f** Evaluations correspond to data from more than 300 cells in each condition: Scramble (*n* = 347) and NOTCH1 KD (*n* = 339) obtained from three independent biological replicates for each condition. Graph bars represent mean ± SEM. Significance was assessed by a Mann–Whitney *U-*test with **P < 0.01. **b** Average number of spikes in each responding cell is greater for NOTCH1 KD compared to Scramble. **c** Average Ca^2+^ spike plotted for each responding cell for Scramble and NOTCH1 KD. Peaks were aligned at *t* = 0 s. **d** Average Ca^2+^ spike duration for each cell shows increased duration for NOTCH1 KD. **e** Average Ca^2+^ spike amplitude was larger for NOTCH1 KD. **f** Average area under the spike for each cell was significantly larger for NOTCH1 KD cells compared to Scramble. **g** Monolayers of HAECs were cultured in normal media with physiological levels of [Ca^2+^] (1.5 mM) or with CaCl_2_ added to increase extracellular [Ca^2+^] to 3 mM for 24 h under flow; scale bar = 20 µm. Gaps (marked in white arrow heads) were only observed in the HAEC monolayers cultured with high extracellular [Ca^2+^]. Gaps were quantified by measuring area uncovered to reveal an increase in gaps for monolayers cultured in higher extracellular [Ca^2+^]. Graph bars represent mean ± SEM, *n* = 5. Significance was assessed by *T-*test **P < 0.01. **h** HAECs were flow-conditioned for 24 h then treated with BAPTA-AM or vehicle and subsequently returned to flow for an additional hour in the presence of DAPT; scale bar = 20 µm. Gaps were quantified by measuring the percent area uncovered to reveal that BAPTA treatment significantly improved junctional stability even in the absence of NOTCH1 signaling. For all imaging experiments, three biological replicates were used for each condition. Graph bars represent average ± SEM. Significance was assessed by *T-*test **P < 0.01

With transcriptome analysis revealing the upregulation of a significant number of intracellular calcium-dependent genes, we measured intracellular [Ca^2+^] changes triggered by the onset of flow, in the presence and absence of NOTCH1 signaling. Confluent monolayers of HAECs loaded with the fluorescent Ca^2+^ indicator Oregon Green BAPTA-1 were monitored for changes in fluorescence intensity under laminar flow. In each experiment, 81–128 individual cells were measured simultaneously with 1 s intervals over 10 min of applied flow (Supplementary Movies 1 and 2). Both groups had comparable resting intracellular [Ca^2+^] levels, 125 and 121 nM, respectively. The majority of endothelial cells responded to the onset of flow with one or more temporary increases in [Ca^2+^], i.e., Ca^2+^ spikes (representative traces in Fig. 7a).

Over the 10 min, the total number of cells that responded to flow was 87% for Scramble and 94% for NOTCH1 KD. The average number of spikes in responding cells was increased in NOTCH1 KD (Fig. 7b). Although the spike profiles were similar (Fig. 7c), further analysis indicated that reduction of NOTCH1 significantly changed the average characteristics (Supplementary Fig. 6a) of the Ca^2+^ spikes: duration, amplitude, and area under the spikes. The average duration of the Ca^2+^ spikes was larger for NOTCH1 KD cells compared to Scramble, 10.7 ± 4.1 vs. 8.6 ± 0.7 s (Fig. 7d). The average Ca^2+^ spike amplitudes were larger for NOTCH1 KD than Scramble, Δ[Ca^2+^] = 7.8 ± 0.6 vs. 4.1 ± 0.3 nM (Fig. 7e) and the average area under each spike in NOTCH1 KD (74 ± 6 nM) was significantly larger for Scramble (33 ± 3 nM) (Fig. 7f and Supplementary Fig. 6b). From these results, we concluded that a reduction in NOTCH1 leads to increased flow triggered Ca^2+^ signaling and hypothesized that this increase in Ca^2+^ signaling underlies the previously observed cell–cell junctional integrity loss under flow. To test this hypothesis, we flow-treated endothelial monolayers cultured in media with high (3 mM) [Ca^2+^] to increase Ca^2+^ signaling for 24 h and compared these with monolayers cultured under flow in physiological (1.5 mM) [Ca^2+^]. Monolayers cultured under physiological conditions had continuous cell–cell junctions, whereas those treated with high [Ca^2+^] displayed gaps between cells (Fig. 7g). Conversely, we set out to determine whether reducing intracellular Ca^2+^ signaling under flow would rescue the cell–cell junctions in the absence of NOTCH1 signaling. For this, we loaded the cell permeable Ca^2+^ chelator BAPTA-AM (BAPTA is trapped inside the cell after cleavage) or vehicle control into flow-conditioned HAECs. After reagent loading, monolayers were subjected to flow in the presence of DAPT to block NOTCH1 signaling and we quantified the integrity of cell–cell junctions by measuring the area uncovered (Fig. 7h). In monolayers with BAPTA-loaded cells, the area uncovered was significantly reduced when compared to monolayers that were not loaded with BAPTA (0.56 ± 0.07% vs 2.65 ± 0.36%, Fig. 7h). Control monolayers were evaluated to eliminate the possibility of secondary effects due to vehicle or BAPTA-AM loading (Supplementary Fig. 6c). In the absence of NOTCH1 signaling, BAPTA-loaded monolayers significantly retained cell–cell junctions to a greater degree than vehicle-treated monolayers (Fig. 7h). This rescue of the NOTCH1 suppression phenotype further indicates that intracellular Ca^2+^ dynamics are modulated by NOTCH1 under flow. Taken together, these findings strongly support our hypothesis that reduced NOTCH1 signaling leads to destabilization of cell–cell junctional integrity through increased Ca^2+^ signaling under flow.

### Loss of endothelial Notch1 in vivo results in inflammation

**Fig. 8:**
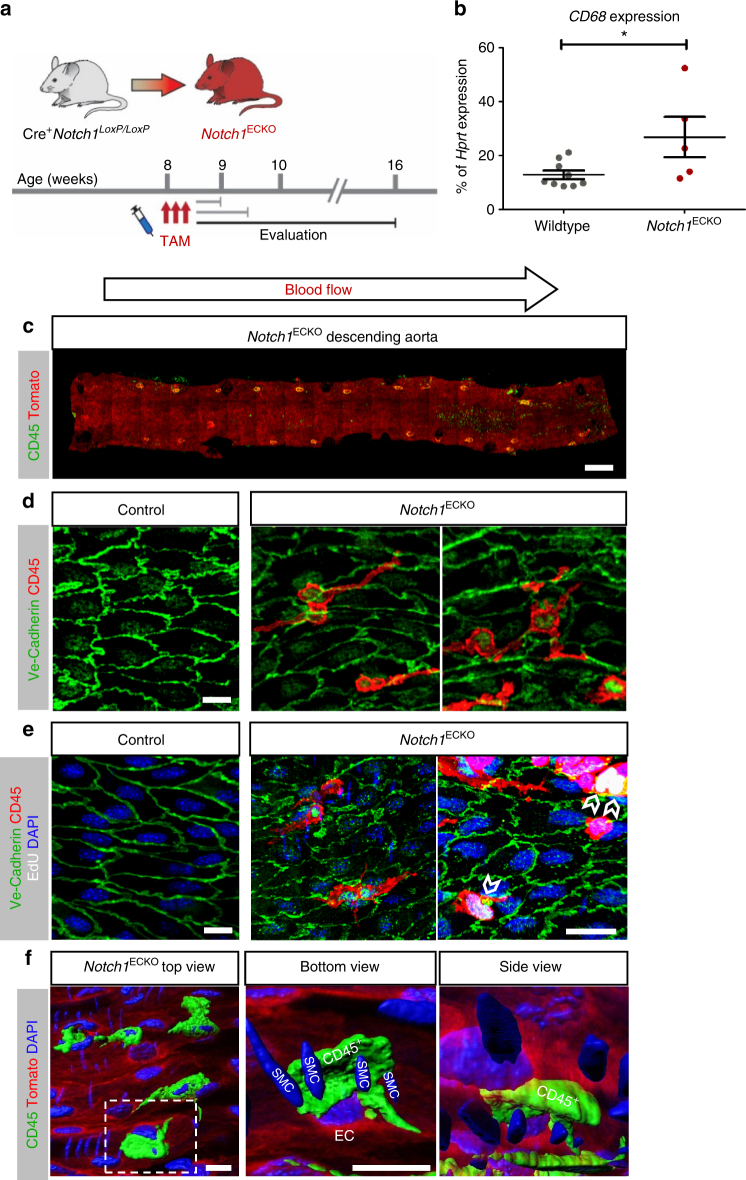
In vivo deletion of endothelial *Notch1* results in inflammatory cell recruitment. **a** Timeline for inducible deletion of endothelial *Notch1* to indicate animal ages and evaluation time points. **b** Quantification of *CD68* expression from the intima of descending aortae of adult WT mice (*n* = 9) and *Notch1* ^ECKO^ mice (*n* = 5) 2 weeks post tamoxifen by qPCR analysis. Graph bars represent average ± SEM, *T*-test *P < 0.01. **c** Confocal tile scan of the descending aorta of *Notch1* ^ECKO^ animal at 8 weeks post tamoxifen reveals the presence of CD45^+^ tdTomato^−^ cells embedded within the tdTomato^+^ endothelium. Scale bar = 500 µm. **d** Ve-Cadherin and CD45 staining of control (Cre^−^ *Notch1* ^loxP/loxP^) and *Notch1* ^ECKO^ descending aortae at 8 weeks post tamoxifen displays CD45^+^ inflammatory cells at higher magnification in the *Notch1* ^ECKO^ aorta; scale bar = 10 µm. **e** En face imaging of the endothelium by Ve-Cadherin staining in the descending aorta of control (Cre^−^ *Notch1* ^loxP/loxP^) and *Notch1* ^ECKO^ mice, both tamoxifen-injected and sacrificed after 8 weeks, shows proliferating (EdU^+^) CD45^+^ inflammatory cells (marked by open arrow heads) embedded within the endothelial layer of *Notch1* ^ECKO^ mice; scale bars = 10 µm. **f** Three-dimensional surface render to visualize the spatial location of marked CD45^+^ cell relative to the endothelium (marked EC) and smooth muscle cells (marked SMC). Both bottom and side view reveal that the CD45^+^ cell is locating above the smooth muscle cell layer. Scale bars = 10 µm

With transcriptome analysis of siNOTCH1 HAECs indicating an enhancement of genes associated with inflammation (Fig. 6a, c) and increase in *CD68* expression in the tunica intima of *Notch1* ^ECKO^ adult mice (Fig. 8b), we further evaluated *Notch1* ^ECKO^ aortas at 8 weeks post deletion (Fig. 8a) to assess the long-term biological consequences of *Notch1* deletion. Imaging of the descending aorta of *Notch1* ^ECKO^ mice 8 weeks post deletion confirmed the presence of CD45^+^ inflammatory cells embedded within the tdTomato^+^ endothelial layer (Fig. 8c). CD45^+^ inflammatory cells were frequently found between endothelial cells in the *Notch1* ^ECKO^ descending aorta, but not in controls (Fig. 8d). The presence of these inflammatory cells was manifested in all descending aortae of *Notch1* ^ECKO^ animals evaluated (*n* = 10). Interestingly, complete blood count (CBC) analysis did not show differences in circulating monocytes, lymphocytes, or neutrophils for *Notch1* ^ECKO^ animals compared to controls at any time point evaluated (Supplementary Fig. 7a, b). Instead, we found that the CD45^+^ inflammatory cells potentially proliferated in situ, as evidenced by EdU incorporation (white open arrow heads in Fig. 8e). To determine the topographical location of the inflammatory cells within the aortic wall, we generated a 3D surface render of the confocal Z-stack images. CD45^+^ cells were found both at the endothelial surface and in the tunica intima (Fig. 8f and Supplementary Fig. 7c, d). Histological sections of aorta and livers of *Notch1* ^ECKO^ animals showed an influx of inflammatory cells in close proximity to the vessel lumen (Supplementary Fig. 7e, f). These data point to the requirement of endothelial Notch1 to limit inflammation.

### Loss of endothelial Notch1 augments atherosclerotic plaques

**Fig. 9:**
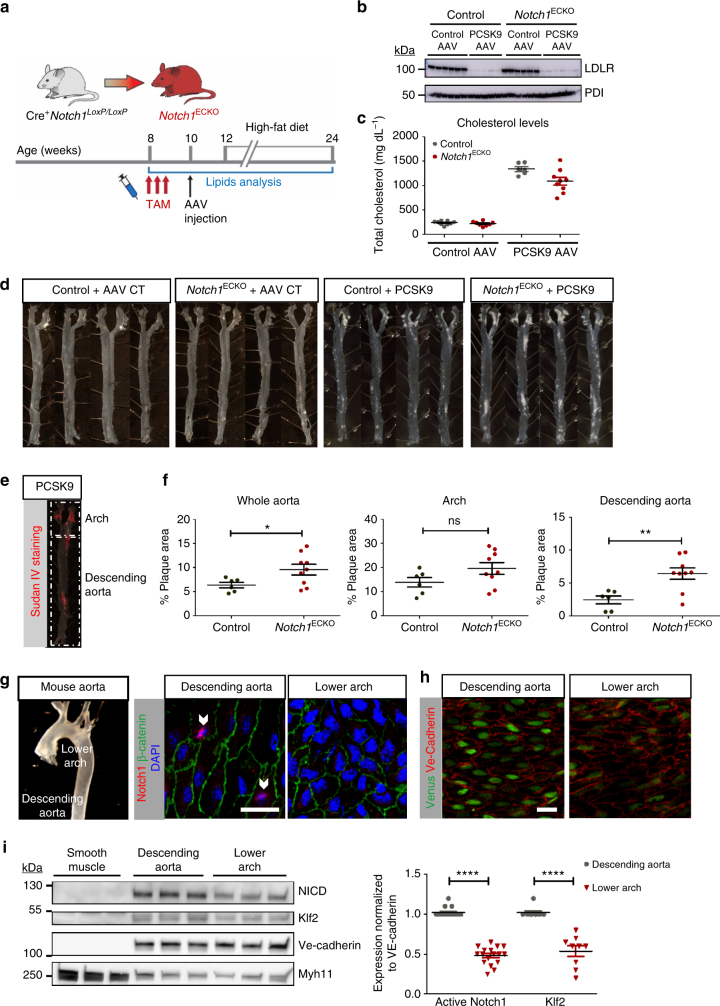
Loss of endothelial Notch1 augments atherosclerotic plaque burden in a model of hypercholesterolemia. **a** Timeline for mouse model of hypercholesterolemia. **b** Quantification of liver LDLR protein levels by immunoblot to confirm PCSK9 activity in PCSK9-AAV-injected animals compared to control-AAV-injected animals at time of harvest (PDI used as loading control). Each lane corresponds to one animal. **c** Circulating cholesterol levels at time of evaluation (12 weeks after AAV injection) (*n* = 6–11 per group, each dot corresponding to one animal). Indicated are individual cholesterol levels in each group. **d** Representative aortas from each of the four groups: Control + AAV CT, *Notch1* ^ECKO^ + AAV CT, Control + PCSK9, and *Notch1* ^ECKO^ + PCSK9. Control animals were Cre^+^ tdTomato reporter. **e** Example of aorta stained with Sudan IV for quantification of atherosclerotic plaque area and identification of the arch and descending aorta regions. **f** Quantification of plaque burden in the whole aorta, arch, and descending aorta was determined by calculating the percent of aortic surface area covered by atherosclerotic lesions in each group (*n* = 6 for Control and *n* = 9 for *Notch1* ^ECKO^). Each point represents the lesion area per mouse. The mean area for each group of mice is indicated by the horizontal bars. *T-*test *P < 0.05, **P < 0.01, ns = not significant. **g** En face imaging of wild-type (C57BL/6) adult mouse aorta reveals enhanced nuclear presence of Notch1 (red) in endothelial cells of the descending aorta compared to endothelial cells of the lower arch (representative images of *n* = 6). Scale bar = 20 µm. **h** Venus Notch reporter mouse aorta imaged en face to assess Notch signaling comparing the descending aorta to the arch (representative images of *n* = 3). Scale bar = 20 µm. **i** Protein lysates from bovine aorta (descending aorta, lower arch and smooth muscle) were analyzed by immunoblot for expression of Notch1 intracellular domain (NICD) and Klf2 using Ve-Cadherin as loading control. Each lane shows isolation from a distinct biological replicate. NICD levels trend with Klf2 expression level differences between the descending aorta and lower arch (data from nine technical replicates isolated from four biological replicates are shown on graph, *T-*test ****P < 0.0001)

To investigate the role of endothelial Notch1 in a pathophysiological setting, we utilized an atherosclerosis mouse model (Fig. 9a) in which hypercholesterolemia was induced via PCSK9-AAV^38^. In brief, 2 weeks post tamoxifen, control and *Notch1* ^ECKO^ animals were injected with PCSK9-AAV or control AAV and subjected to a high-fat diet for 3 months. Throughout the study and every 2 weeks, animal body weights were measured and blood was drawn via retro-orbital bleed to assess circulating lipid levels. At the time of termination, liver protein lysates were used to determine LDLR protein by immunoblot (Fig. 9b). A significant increase in total cholesterol was evident in animals injected with PCSK9-AAV, both control and *Notch1* ^ECKO^ animals (Fig. 9c). No difference in body weight was found between control and *Notch1* ^ECKO^ animals at 4 weeks post tamoxifen (Supplementary Fig. 8a). Analysis of animals injected with control AAV over the 3 months of high-fat diet, exhibited a slight trend toward increased body weight for *Notch1* ^ECKO^ animals at time of harvest (Supplementary Fig. 8b). Animals injected with PCSK9 showed the sustained increase in total cholesterol (Fig. 9c and Supplementary Fig. 8c), but no significant difference in body weight (Supplementary Fig. 8d). Atherosclerotic lesions were observed in all animals injected with PCSK9, however no lesions were found in animals injected with control AAV (Fig. 9d showing four aortas from each group). Sudan IV staining was applied to aortas (Fig. 9e) to quantify plaque burden as a percentage of aorta area, defining the upper region as the *Arch* and the lower region as the *Descending Aorta* (marked in dashed boxes in Fig. 9e). Quantification of the plaque area revealed a significant increase in plaque burden for the whole aorta and the descending aorta, but not the arch, for *Notch1* ^ECKO^ animals (Fig. 9f).

To further understand the differential outcomes in aortic plaque burden in the descending aorta vs. arch, we probed Notch1 expression in different regions of the aorta (regions marked in Fig. 9g), representing two extreme (low and high) regions of time-averaged shear stress. We found higher frequency of endothelial cells with nuclear Notch1 staining in the descending aorta compared to the lower arch (arrowheads in Fig. 9g). Notch1 activity in the two regions was further assessed using the Notch reporter mouse and mean fluorescence intensity showed a 1.6-fold increase in the descending aorta compared to the lower arch, indicating that Notch activity was amplified in regions of higher shear stress (Fig. 9h). These findings were corroborated biochemically using endothelial-enriched protein lysates isolated from bovine aortas (Supplementary Fig. 8e). Expression levels of Notch1 intracellular domain (NICD) and Klf2 were quantified using Ve-cadherin as a loading control^39^, and we found that NICD levels followed the same trend as Klf2, a recognized indicator of shear stress^40,41^ with a two-fold enhancement in the endothelium of the descending aorta (Fig. 9i; original gels in Supplementary Fig. 9). Comparing lower arch to upper arch revealed a similar trend in Notch1 activity, with more NICD in the upper arch where shear stress magnitude is higher (Supplementary Fig. 8f). To probe for the response of NOTCH1 signaling under different types of flow, we compared endothelial monolayers cultured statically or under atheroprotective flow under disturbed flow. NOTCH1 gene expression was enhanced under atheroprotective flow, but not under disturbed flow (Supplementary Fig. 8g). Additionally, the NOTCH1 target *HES1* was upregulated under atheroprotective, but not under disturbed flow, following the same trend as *KLF2*. Investigation of inflammatory gene expression changes under atheroprotective flow revealed an increase in *ICAM1*, *IL8* and *CXCL2* in NOTCH1 KD monolayers (Supplementary Fig. 8h). Interestingly, the suppression of NOTCH1 did not alter *ICAM1*, *IL8*, or *CXCL2* gene expression under disturbed flow where average shear stress is very low (Supplementary Fig. 8i). Combined, these data provide evidence that endothelial NOTCH1 signaling correlates positively with the level of shear stress in the aorta in vivo and that the presence of Notch1 in the descending aorta protects the endothelium from atherosclerotic plaque build-up in the advent of hypercholesterolemia.

## Discussion

Responses of endothelial cells to laminar flow include alignment, suppression of proliferation, and acquisition of an anti-inflammatory profile^42,43^. While these phenotypes are recognized landmarks of cells experiencing laminar flow, the mechanisms involved in promoting these effects remained unclear. Here we demonstrate that NOTCH1 signaling is required for all three phenotypes. Reduction of NOTCH1 in vitro or genetic inactivation in vivo results in destabilization of cell–cell junctions and continuous endothelial proliferation irrespective of the presence of flow. Intriguingly, classical markers of flow, like KLF2, appear unaffected by deletion of *NOTCH1*. The fact that KLF2 is upregulated by flow in the absence of NOTCH1, supports the concept that KLF2 alone is not sufficient to regulate flow responses associated with junctional stability and cell proliferation. For those actions, NOTCH1 is a critical mediator.

During angiogenesis, Notch1 signaling is essential for tip-stalk specification within vascular sprouts^44^ and to facilitate junctional rearrangements^45^. Later, Notch1 is required for arterial fate^26,27,46^, nonetheless the relevance of Notch1 in adult arterial endothelium is unclear. In vitro, NOTCH1 signaling has been reported to increase with shear stress^22,23^, yet activation of Notch1 by flow in vivo has been disputable with findings supporting a direct effect^23^ and others indicating that Notch1 is suppressed by shear stress^47,48^. A critical aspect that might explain these discrepancies relates to the type of flow, endothelial cell type used (lymphatic vs. blood^47^), levels of shear stress, and stage in development. Here we demonstrate that only laminar flow increases NOTCH1. Furthermore, we also show that NOTCH1 responds exquisitely to the level of shear stress. In fact, using flow velocity gradients, we demonstrated a highly sensitive direct positive relationship between shear stress and NOTCH1 signaling in real time. Our findings also revealed that NOTCH1 protein is polarized downstream of flow direction regardless of cellular polarity, as indicated by the direction of cell migration in arterial wounds. By changing flow direction in vitro, we further uncovered that this relationship is dynamic and physiologically tuned to its environment with rapid relocation of the protein. Combined, our findings indicate that NOTCH1 pathway is able to sense and respond to physical flow forces. Importantly, NOTCH1 activation requires forces on the order of 12pN^24^, and therefore constant alterations in cellular tension are likely important contributors to its activation.

As to proliferative responses, it has been reported that NOTCH1 is activated by cell–cell contact under static conditions to promote cell cycle arrest^49,50^. Our data are fully consistent with those in vitro findings and further support the notion that utilization of the NOTCH1 pathway by flow activation is a mechanism by which cell cycle can be titrated and vascular homeostasis preserved. In the absence of NOTCH1 this suppression is muted, regardless of the presence of flow, as demonstrated here by EdU incorporation in the aortic endothelium of *Notch1* ^ECKO^ mice and presence of mitotic figures. The profile associated with proliferation is robust and suggests that Notch1 suppresses a default state that can actively promote cell cycle entry. Genes found to be transcriptionally increased in the absence of NOTCH1 include cyclins and cdks and several regulators of cell cycle (Supplementary Table 1). This is a vigorous program that clearly shows a commitment to cell division. We also found that transcripts for *EFNB2*, a marker of arterial identity, are reduced when NOTCH1 is suppressed. The data indicate that even in the adult, NOTCH1 is required to constantly maintain EPHRIN B2, and therefore, arterial identity.

Exploration of the mechanisms by which NOTCH1 signaling might rapidly and concurrently affect junctional stability, cell cycle, and elongation driven by flow were elusive until we noted a link between NOTCH1 and calcium homeostasis. Reduction of NOTCH1 reveals an emerging signature of calcium-regulatory genes. These include CAMK2B a calcium/calmodulin-dependent protein kinase involved in multiple functions including regulation of Ca^2+^ release from the endoplasmic reticulum^51^; Apelin that, amongst other functions, was shown to regulate endoplasmic reticulum calcium channels (SERCA and ryanodine receptors)^52^; Connexin-37, involved in the formation of gap junctions that increase intercellular transport of calcium; and Aconitase, an enzyme that in addition to regulating tricarboxylic acid cycle also contributes to connexin hemichannel opening^53^. In total, 14 different transcripts associated with calcium regulation were increased upon NOTCH1 suppression (Supplementary Table 3). The transcriptional signature implies that NOTCH1 is involved in the rapid regulation of intracellular calcium, although it remains unclear whether these are direct NOTCH1 targets and whether these effects are mediated by a canonical NOTCH1 signaling event. Nonetheless suppression of NOTCH1 results in the increase of intracellular calcium signaling under flow. Importantly calcium is a powerful second messenger and slight increases in cytosolic levels have been associated with cell cycle regulation^54–56^, cell–cell junctions^57^, focal adhesion, and cytoskeletal changes^58,59^. The emerging concept from these findings is that NOTCH1 might be a modulator of intracellular calcium spikes and associated signaling events that are induced by flow^60–62^. In fact, recent work showed that Ca^2+^ oscillations were increased in tip cells vs. stalk cells during sprouting angiogenesis in zebrafish^63^. We propose that blood flow adjusts NOTCH1 to proportionally regulate Ca^2+^ signaling to ensure homeostasis.

Finally, we also showed that NOTCH1 signaling regulates several pro-inflammatory genes associated with atherosclerosis. The list included genes involved in recruitment of inflammatory cells such as *CXCL2*, *ICAM1*, *CXCR4*, and several *CXCL* ligands and interleukins (Supplementary Table 2). The list of transcripts reveals that NOTCH1 contributes to suppress endothelial binding to inflammatory cells and to enhance self-immunity. In the absence of NOTCH1, inflammatory cells incorporate within the endothelial layer and transmigrate across the intima. Of note, we find that *VCAM* is a direct target of NOTCH1 and it is suppressed rather than induced when NOTCH1 is reduced (Supplementary Table 2). The binding of macrophages is likely facilitated by intercellular gaps, as accumulation of fibrin most certainly enables binding and further spreading of these cells. These results are consistent with our finding that *NOTCH1* haploinsufficiency in the context of a pro-atherogenic genetic background increases atherosclerosis in the aorta^21^ and further reveal that the protective role of NOTCH1 is particularly associated with the areas of laminar flow.

Overall, this study fills important gaps in our understanding of how blood flow controls endothelial responses. The data presented show that increases in laminar flow are sensed by NOTCH1 resulting in polarization and nuclear NICD translocation. Importantly, while endothelial cells are still able to sense flow, as per KLF2 increase, absence of NOTCH1 impairs their ability to remain mitotically quiescent and maintain junctional integrity. The pathophysiological consequences include increased susceptibility to atherosclerosis in areas considered to be atheroprotective. In this manner, NOTCH1 might be the point of conversion that interprets flow levels and controls responses to potential pathological conditions.

## Methods

### Cell culture

HUVECs (VEC Technologies #HUVEC/T-75 Flask), HAECs (Lonza #CC-2535 and ATCC # PCS-100–011), and BAECs (isolated from bovine aortas) were cultured in complete MCDB-131 medium (VEC Technologies; MCDB131-WOFBS) with 10% fetal bovine serum (Omega Scientific #FB-11). All primary endothelial cells were checked for mycoplasma and cultured on gelatin (Fisher Scientific # NC9369923) coated dishes in humidified incubator at 37 °C and 5% CO_2_ and used between passages 4 and 9.

### Shear stress

Endothelial cells were seeded in ibidi µ-Slide y-shaped ibiTreat chambers (ibidi #80126) or µ-Slide I 0.4 Luer ibiTreat chambers (ibidi #80176) and unidirectional flow was applied after confluent monolayer reached using the ibidi pump system (ibidi #10902). For RNA and protein lysis, laminar shear stress was applied using a modified cone and plate device^64^ consisting of a computerized stepper motor UMD-17 (Arcus Technology #DMX-UMD-17-2) and a 1° tapered stainless steel cone set at 700 µm height above the plate. The flow devices were maintained in 37 °C incubator with 5% CO_2_. Confluent endothelial monolayers were grown on gelatin-coated six-well plates (Falcon #08-772-1B) in complete MCDB-131 media (VEC Technologies # MCDB131-WOFBS) plus 10% FBS (Omega Scientific #FB-11) containing 4% dextran (Sigma-Aldrich #31392). Monolayers were subjected to unidirectional constant laminar flow, pulsatile laminar flow (termed atheroprotective), or disturbed flow^15^ for set time points before lysates were harvested, at which point the center of each well was removed with a cell scraper (BD Falcon #35-3085) before lysis buffer was added. Static monolayers used the same dextran used the same dextran-containing media and were cultured alongside flow-treated monolayers. For pre-aligned treatments, DMSO or DAPT were added after 48 h of flow treatment directly to culture medium within the well.

### Immunofluorescence and imaging

Cells were fixed in flow chambers with 2% PFA or methanol (Sigma-Aldrich #322415) for 10 min followed permeabilized with 0.1% Triton X-100 (Sigma Aldrich #X100) in 1× PBS and blocked for 2 h with 5% Normal donkey serum (Jackson Immuno Research Laboratories #017-000-121) in 1X PBS. Primary antibodies were incubated overnight at 4 °C in blocking buffer and secondary antibodies applied for 2 h at room temperature. To harvest mouse aortas for imaging, the animals were injected with 6.5 mg methacholine chloride (MP Biomedicals # 0219023105) dissolved in 1× PBS to relax smooth muscle and then perfusion-fixed with 2% PFA via left ventricle. The aorta was subsequently dissected from the spine, adventitia removed, cut longitudinally, and then pinned opened in silicone-coated dishes for staining after further fixation, permeabilization, and block. The aortas were mounted en face using ProLong Gold Antifade Mountant (Fisher Scientific #P36930). Primary antibodies: anti-β-catenin rabbit polyclonal (1:200; Sigma #C2206), anti-β-catenin goat polyclonal (C-18) (1:200; Santa Cruz Biotechnology #sc-1496), anti-NOTCH1 (D1E11) rabbit monoclonal (1:200; Cell Signaling #3608), anti-NOTCH1 ECD mouse monoclonal (1:200; BD Biosciences #MHN1-519), anti-VE-cadherin goat polyclonal (Santa Cruz Biotechnology #sc-6458), anti-Giantin mouse monoclonal (1:200; Abcam #ab37266), anti-acetylated tubulin mouse monoclonal (1:200; Sigma #T7451), anti-GFP chicken polyclonal (1:200; Abcam #ab13970), anti-ERG (EPR3864) rabbit monoclonal (1:200; Abcam #ab92513), anti-mouse CD45 Clone 30-F11 rat monoclonal (1:200; BD Biosciences #550539), and anti-NFAT2/NFATC1 rabbit polyclonal (1:200; Novus Biologicals #NB100-56732). Fluorescently tagged secondary antibodies were purchased from Invitrogen, applied at 1:400 dilution and in some instances used in combination with Texas Red-X Phalloidin (1:40; Life Technologies # T7471) and DAPI (1:1000; Thermo Scientific #62248). For mouse aorta *en face* imaging of Notch1, Tyramide Signal Amplification (TSA) kit (ThermoFisher Scientific #T20934) was used and followed according to the manufacturer’s supplied protocol. The images were captured with ×10, ×20, or ×40 objective on a Zeiss LSM710 or LSM880 confocal microscope with ZEN software (Carl Zeiss Microscopy) for acquisition. Image processing and quantification of parameters was performed with Imaris software (Bitplane) or ImageJ.

### Transfection and γ-secretase inhibition

Confluent monolayers of endothelial cells were transfected with stealth RNAi targeting NOTCH1 (NM_017617.3_Stealth_775: Sense-GAC GAU UGU CCA GGA AAC AAC UGC A and Antisense-UGC AGU UGU UUC CUG GAC AAU CGU C) or control Stealth RNAi™ siRNA Negative Control, Med GC (Invitrogen #12935300) using siPORT *Amine* transfection agent (Invitrogen #AM4503) in Opti-MEM® Reduced Serum Medium (Gibco # 31985070) without antibiotics. For improved efficiency, the cells were transfected twice with 24 h of recovery in between; cultures were then used for experiments within 12 h after the second transfection. For in vitro NOTCH signaling experiments, Cignal RBP-Jk Reporter (GFP) kit (QIAGEN #CCS-1014G) was used by transfecting HUVECs or HAECs according to the manufacturer’s guidelines. For experiments involving γ-secretase inhibition, 10 or 50 µM InSolution γ-secretase inhibitor IX (EMD Millipore #565784) or equivalent volume of dimethyl sulfoxide (DMSO) (Hybri-Max™, sterile-filtered, BioReagent ≥99.7% Sigma-Aldrich #D2650) was applied to the cell culture medium for the duration of the experiment.

### Western blot analysis

Endothelial cells were lysed in modified RIPA buffer containing 1% Triton X-100 and 10% SDS. For all in vitro cultures, the endothelial cell monolayers in 6-well plates were lysed after removing the center region using cell scraper (BD Falcon #35-3085) and washing with 1X HBSS (Corning #21-022-CV) to avoid the region not subjected to uniform shear stress. Flow-treated cultures and static controls were handled equivalently. Proteins were separated by SDS-PAGE gradient (4–20%) gel and transferred onto nitrocellulose membranes and incubated overnight at 4 °C with primary antibodies. Primary antibodies: anti-Ve-Cadherin rabbit polyclonal (1:1000; Cell Signaling #2158), anti-NOTCH1 (D1E11) rabbit monoclonal (1:1000; Cell Signaling #3608), Cleaved NOTCH1 (Val1744) (D3B8) rabbit monoclonal (1:500; Cell Signaling Technology #4147), anti-Klf2 rabbit polyclonal (1:500; EMD Millipore #09-820), anti-Cyclin D1 rabbit monoclonal (1:500; Abcam #ab16663), anti-gamma H2A.X (phospho S139) rabbit polyclonal (1:1000; Abcam #ab11174), anti-smooth muscle Myosin heavy chain 11 rabbit polyclonal (1:1000; Abcam #ab53219), anti-gamma Tubulin rabbit polyclonal (1:2000; Abcam #ab11321), anti-LDL Receptor rabbit polyclonal (1:1000; Cayman #10007665), and anti-PDI rabbit monoclonal (1:10,000; Cell Signaling #3501). HRP-conjugated secondary antibodies (1:10,000) were applied in species dependent manner at room temperature for 1 h. Immuno-complexes were detected by enhanced chemiluminescence with SuperSignal™ West Femto Maximum Sensitivity Substrate (ThermoFisher Scientific #34095) using ChemiDoc XRS + Molecular Imager (Bio-Rad Laboratories). Quantification of bands by densitometry analysis was performed using ImageLab Software (Bio-Rad Laboratories).

### MRI flow mapping

The Y-slide was positioned in a 40 mm i.d. 400 MHz 1H Millipede vertical imaging probe within 9.4T Varian microimaging system. Flow was imaged using a spin-echo multi-slice (SEMS) pulse sequence modified for phase-contrast MRI velocimetry^65^. Briefly, all gradients in the SEMS pulse sequence, except for the phase encoding gradient, were appended with flow-compensation gradients. Bipolar, trapezoidal, FW gradients were added in the *x*, *y* and *z* directions to select the gradient first moment, M1. The residual phase accumulation from each F.W. gradient is proportional to the fluid velocity in the respective direction when M1 is properly chosen to avoid phase wrapping^66^. In the case of no flow, phase accumulation of static nuclear spins resulting from a gradient value of +M1 and −M1 is neutralized, resulting in a zero net phase accumulation. Fluid flow speeds were calculated using the *x*, *y* and *z* velocity components. Flow vector maps were generated from the *x* and *y* velocity components. To compensate for directional fluid velocity bias, the y-slide was imaged, physically rotated 180° about the *x* axis inside the bore of the magnet, and imaged again. The initial position and 180° rotated F.W. images were averaged together to generate the final result. To account for gradient imperfections, each of these images was generated by subtracting a flow-weighted image of the slide in the absence of flow. Corresponding shear maps were determined using the equation: $\tau _w = \frac{{6\mu Q}}{{bh^2}}$ where $\tau _w$ is the shear stress at the endothelial cell monolayer, *µ* is the dynamic viscosity of cell culture media at 37 °C, *Q* is the volumetric flow rate through a voxel, *b* is the width of a voxel, and *h* is the height of the ibidi chamber. This equation is derived from the Navier–Stokes equation^67^, given a condition of laminar flow between two infinitely wide parallel plates.

### NOTCH1 protein polarization kinetics

To evaluate the spatial distribution of NOTCH1 intracellular domain (ICD) and extracellular domain (ECD), after flow alignment for 48 h, HAECs were exposed to reverse flow direction for different periods of time. For each time point of reverse flow, at least three biological replicates were used from which *n* ≥ 116 individual cells were measured. To estimate the concentrations of NOTCH1 ICD and ECD, ImageJ was used to calculate the fluorescence along the cell body length and parallel to the flow axis. Cell bodies that were partially visible were discarded. Using the center of the nucleus as a point of reference, the distance from both ends of the cell body to the center of the nucleus were normalized to 1, allowing the evaluation of the distribution of ICD and ECD as a function of time and position along the cell body. To evaluate the concentration of ICD and ECD in each side of the cell bodies with respect to the nucleus, we removed the background fluorescence and evaluated the area under the curve of fluorescence in each side separately and normalized by both areas. To evaluate the polarization kinetics, the average concentration of ICD and ECD at each time point analyzed was then fitted with an exponential function and measured the diffusion rate as the exponential decay’s characteristic time.

### Intracellular calcium imaging

HAECs were plated on y-slide (ibidi #80126) and after reaching confluence, cells were treated with 15 µM Oregon Green 488 BAPTA-1-AM ester (OGB-1-AM) (Thermo Fisher Scientific #O6806). The AM ester can freely pass the cell membrane; but once inside, the AM is cleaved by esterases leaving OGB-1 trapped in the cell. OGB-1-AM was reconstituted in Pluronic F-127 (Invitrogen #P-3000MP) and diluted in phenol-red-free MCDB-131 (VEC Technologies #MCDB-131 WOFBSPR) with 10% FBS (Fisher Scientific, Cat. No. MT 21-023-CV). After 20-min incubation with OGB-1-AM, the cells were washed with 1 µg/mL Hoescht (Thermo Fisher Scientific #PI-62249) diluted in MCDB-131 (MCDB-131 WOFBSPR) and incubated for an additional 20 min. Slides were then connected to the yellow/green perfusion set (ibidi #10963) modified with non-permeable tubing containing 13 mL of conditioned MCDB-131 (MCDB-131 WOFBSPR, phenol red free). All steps were completed with minimal light exposure. After beginning flow (20 dynes cm^−2^), fluorescence images were acquired using Zeiss Observer Z1 with Colibri 7 light source, ORCA-Flash4.0 camera (Hamamatsu #C13440-20CU) and ZEN 2 (blue edition) software. Images were acquired once every second with a 50 ms exposure. After 10 min of imaging the flow was stopped. To obtain *F*_max_, the calcium ionophore ionomycin (Life Technologies #I24222) was added to the perfusion set to obtain a final concentration of 1.5 µM. Flow was restarted, and images were captured every second for 3 min. At the conclusion of 3 min, the flow was stopped and media in perfusion reservoirs removed. To obtain *F*_min_, conditioned HEPES buffered EDTA solution (20 mM EDTA, 120 mM NaCl, 5 mM KCl, and 10 mM HEPES) was added, the flow was restarted and images were captured every second for 3 min.

### Calcium imaging analysis

To detect and segment cells, all frames of the calcium imaging videos were averaged and Otsu’s threshold applied. To remove photo bleaching from the calcium imaging, a non-linear fitting of the signal was used to subtract the slow decay. Because the signals often consisted of several wide peaks that represented a reasonable portion of the time series, an asymmetric loss function was used to penalize overestimations more than underestimations. The fitted model combined a second degree polynomial with an exponential decay. The asymmetric loss function used in this non-linear fitting was

$$L\left( {y,\hat y} \right) = \left( {y - \hat y} \right)^2\left( {sign\left( {y - \hat y} \right) + \alpha } \right)^4,$$

where *y* represents the predicted values by the fitted model, $\hat y$ represents the actual measured values, and *α* > 0 is a meta-parameter optimized for each sample (for most replicates, *α* ≈ 0.8). The optimization was performed by the Levenberg–Marquard algorithm (lmfit implementation) with the linear coefficient and the exponential characteristic time both constrained to negative values. For more robust fitting, we also added an L2 regularization term on the coefficient of second degree term. After removing the photo bleaching, the calcium concentration was estimated by using the formula *F*_Ca_(*t*) = *K*_d_ (*F*(*t*) − *F*_min_)/(*F*_max_ − *F*(*t*)), where *F*(*t*) is the fluorescence as a function of time^68^, *K*_d_ = 170 nM is the dissociation constant of OGB-1, and *F*_max_ and *F*_min_ were both determined for each cell separately (see above). Figure 7a shows representative time series plots of the calcium concentration *F*_Ca_(*t*) for control cells and for Notch1 KD cells. To detect spikes, a threshold for each replicate was defined in order to select spikes that were two standard deviations above the noise and lasted for at least three seconds. Spike duration for each cell was calculated by measuring how long the calcium concentration *F*_Ca_(*t*) was above a set threshold value. To evaluate the area under the spike, we subtracted from *F*_Ca_(*t*) its minimum and evaluated the total area under the curve. Spike amplitude was measured as the highest calcium concentration, with respect to the baseline, achieved in each spike for each cell.

### Intracellular calcium blocking experiments

HAECs were plated on a Y-shaped slide (ibidi #80126). After reaching confluence, cells were exposed to flow (20 dynes cm^−2^) for 48 h using red perfusion set (ibidi #10962) containing 13 mL of conditioned MCDB-131 with 10% FBS. After 48 h, the cells were treated with 20 µM BAPTA-AM (Life Technologies #B6769) for 20 min. BAPTA-AM was reconstituted in Pluronic F-127 (Invitrogen #P-3000MP) and diluted in MCDB-131 (VEC Technologies #MCDB-131 WOFBS) with 10% FBS (Fisher Scientific, #MT 21-023-CV). After a 20-min incubation with BAPTA-AM, slides were reconnected to perfusion sets and exposed to flow (20 dynes cm^−2^) for 1 h in the presence of 50 µM DAPT or equivalent volume of DMSO. After 1 h, the slides were removed and fixed with 2% PFA and subsequently imaged for confocal microscopy.

### Transcriptional analysis

Endothelial RNA-enriched fractions from mouse aortae were harvested from descending aortae after careful dissection under a dissecting microscope (Zeiss Stereo Discovery V12). The lumen of the aorta was subsequently flushed with 100 µL of RLT lysis buffer (QIAGEN #79216) containing 1% β-mercaptoethanol (MP Biomedicals #0219483483) using an insulin syringe (BD #328431)^69^. Endothelial cell monolayers under flow were also lysed using RLT lysis buffer with 1% β-mercaptoethanol after the center region was removed with cell scraper (BD Falcon #35-3085) and washed in 1X HBSS. Total RNA from cell culture or mouse aortae was purified using the RNeasy Mini kit (QIAGEN #74106). Complementary DNA synthesis was performed with Superscript III reverse transcription First-Strand synthesis kit (Invitrogen #18080051) using oligo-dT primers. qRT-PCR was performed using primers designed for human or mouse targets, each reaction was run in duplicate and gene expression was normalized with the housekeeping gene (HPRT) and relative expression calculated using the ΔΔCt method. Primer sequences for mouse targets: *Hprt* 5′-CTGGTTAAGCAGTACAGCCCCAA-3′ (forward) and 5′-CGAGAGGTCCTTTTCACCAGC-3′ (reverse); *Notch1* 5′-CCCTTGCTCTGCCTAACGC-3′ (forward) and 5′-GGAGTCCTGGCATCGTTGG-3′ (reverse); *Cd68* 5′- GACCTACATCAGAGCCCGAGT-3′ (forward) and 5′-CGCCATGAATGTCCACTG-3′ (reverse). Primers sequences for human targets: *HPRT* 5′-GCCCTGGCGTCGTGATTAGT-3′ (forward) 5′-AGCAAGACGTTCAGTCCTGTC-3′ (reverse); *NOTCH1* 5′-ACTGTGAGGACCTGGTGGAC-3′ (forward) 5′-TTGTAGGTGTTGGGGAGGTC-3′ (reverse); *HES1* 5′-TCA ACA CGA CAC CGG ATA AA-3′ (forward) 5′-TCA GCT GGC TCA GAC TTT CA-3′ (reverse); *KLF2* 5′-CATCTGAAGGCGCATCTG-3′ (forward) 5′-CGTGTGCTTTCGGTAGTGG-3′ (reverse); *ITGB1* 5′-TTATTGGCCTTGCATTACTGCT-3′ (forward) 5′-CCACAGTTGTTACGGCACTCT-3′ (reverse); *PECAM1* 5′-GAAACCATGCAATGAAACCA-3′ (forward) 5′-GACAGCTTTCCGGACTTCAC-3′ (reverse); *ICAM1* 5′-ACC GGA AGG TGT ATG AAC TG-3′ (forward) 5′-AGC GTA GGG TAA GGT TCT TG-3′ (reverse); *IL8* 5′-AAG AAA CCA CCG GAA GGA AC-3′ (forward) 5′-ACT CCT TGG CAA AAC TGC AC-3′ (reverse); *CCL2* 5′-CAG CCA GAT GCA ATC AAT GCC-3′ (forward) 5′-TGG AAT CCT GAA CCC ACT TCT-3′ (reverse). Primer sets were synthesized by Integrated DNA Technologies, Inc.

### NanoString transcriptional analysis

For NanoString gene expression analysis, probe sequences for NOTCH1, HES1, NRARP, FABP4, EFNB2, KLF2, KLF4, and CCND1 were custom designed and manufactured by NanoString Technologies Inc., including sequences of 4 housekeeping genes (HPRT1, TLK2, TUBG1, and USP39) for data normalization, as well as negative and positive controls. Validation of RNA sample quality was done using the BioAnalyzer (Agilent Technologies). HAECs were used for all sample conditions and each culture condition was performed with *n* = 5. The samples were loaded at 100 ng mRNA per well and data collected as absolute counts; detectable levels of non-specific binding (background) were measured by six negative controls and the means plus two standard deviations were subtracted from each count. Analysis of the nCounter Gene Expression CodeSet was performed via nSolver analysis software (NanoString Technologies, Inc.) to calculate fold change expression.

### Transcriptome analysis

Microarray results used for the analysis were deposited in the National Center for Biotechnology Information Gene Expression Omnibus database under accession no. GSE72633. HAECs were transfected in triplicate with stealth RNAi targeting NOTCH1 (NM_017617.3_Stealth_775) or control duplexes (Invitrogen) using siPORT Amine (Ambion) as the transfection agent. Cells were transfected twice with 24 h of recovery between, cultures were then used for experiments 24 h after the second transfection. For each siRNA condition, three biological replicates were performed and evaluated. Total RNA samples with RNA integrity numbers (RIN) of 7.0 or higher were hybridized to Illumina Human HT-12 v4 Expression BeadChips (San Diego, CA), which contained 48,804 expression and 786 control probes. Differential gene expression analyses to compare cells transfected with siRNA against NOTCH1 or scrambled siRNA was performed using the limma package in R (v2.13.0) with non-parametric background correction followed by quantile normalization. Calculated *P*-values from the moderated *t*-statistics were adjusted using the Benjamini–Hochberg method for multiple testing. Microarray probes with adjusted values of *P* < 0.01 (1% FDR) and fold change > 50% were considered differentially expressed. For visualization of the differential gene expression log-2-fold change was plotted against −log10 of *P*-value using R and points were color coded based on gene ontology and defined fold change cut-offs.

### Lipid analysis

Total plasma cholesterol was determined using an enzymatic, colorimetric assay kit (Infinity Cholesterol; Thermo Fisher), according to manufacturer’s instructions. Briefly, plasma was isolated by centrifugation and stored at −80 °C until assaying. For each assay plate, a standard curve ranging between 0 and 300 mg dL^−1^ was run. A volume of 10 μL of standard or sample was pipetted onto a 96-well plate and 200 μL of working reagent (Infinity Cholesterol) added. The plates were covered, incubated for 15 min, and read at 550 nM (with a reference wavelength of 650 nM). All samples were performed in triplicate, where any replicates differing by 5% or more were re-assayed.

### Animal experiments

Animal protocols were reviewed and approved by the UCLA Institutional Animal Care and Use Committee. C57BL/6J mice were purchased from Jackson Laboratory (stock #000664) to expand the colonies, both female and male adult mice were used in all the experiments. For inducible endothelial deletion of *Notch1*, *Cdh5*(PAC)-CreERT2 (Tg(*Cdh5*-cre/ERT2)1Rha) mice, *Notch1* ^tm2Rko^ loxp line were crossed with R26RTd Cre reporter lines (*Gt*(ROSA)*26Sor* ^tm14(CAG tdTomato)Hze^)^70^. Eight-week-old mice received intraperitoneal injections of tamoxifen (1 mg per mouse; MP Biomedicals #02156738) for three consecutive days and then sacrificed as indicated. CBF:H2B-Venus reporter mice (Tg(Cp-HIST1H2BB/Venus)47Hadj/J) were purchased from the Jackson Laboratory (stock #020942) and used for imaging experiments. To quantify proliferation, EdU (5-ethynyl-2′-deoxyuridine) (Invitrogen #A10044) was applied via intraperitoneal injection (1 mg in 1× PBS warmed to 37 °C) 2 h before animal sacrifice. Click-iT® EdU Alexa Fluor® 488 Imaging Kit (Invitrogen # C10337) was used on 2% PFA fixed aorta and followed according to the manufacturer’s protocol.

For the mouse hypercholesterolemia study, AAV-PCSK9 and control was a gift Dr. H. Jo^38^. Two weeks after tamoxifen injections, the mice were injected with AAV-PCSK9 (1 × 10^11^ VG) or control AAV via tail vein and fed a high-fat diet (16% fat and 1.25% cholesterol, Research Diets #D12336) for 3 months. Blood was collected every 2 weeks via retro-orbital bleed to assess circulating lipid levels. At the time of harvest, livers were isolated to obtain protein lysates and the aortas were subsequently perfused fixed and stored in 4% PFA + 7.5% sucrose. Aortas were then pinned on black wax, rinsed in 70% ethanol for 5 min, stained with Sudan IV solution for 15 min, and de-stained in 80% ethanol. Images were taken using Zeiss Stereo Discovery V12 microscope with Zeiss Axiocam HRC and Axiovision software. To automate the aortic plaque quantification, ImagePro Premier software (Media Cybernetics) was used to apply Spatial Calibration and Smart Segmentation tools to determine the total aorta area and plaque area. The plaque area was quantified as a percent of the total aorta and as a percent for the arch, defined by the arch and 3mm from the arch bifurcation cut, and the descending aorta, defined by the remaining aorta length.

Mouse experiments were conducted in accordance with UCLA Department of Laboratory Animal Medicine’s Animal Research Committee guidelines.

Bovine aortas were obtained from Sierra for Medical Science (Whittier, CA) and from animals that were sacrificed for purposes other than experimental.

### Statistics, sample size, and blinded analysis

Statistical analyses were included in almost all of the experiments with the few exceptions of qualitative image analysis. Dr. David Elashoff (biostatistics core at UCLA) provided oversight. Determination of variance between groups, parametric, or non-parametric analysis was assessed and either *t*-test or Mann–Whitney was applied to determine significance. Most analysis were performed using Prism software (GraphPad Software). *P* < 0.05 was considered significant and reported to the graphs. Data are represented as mean ± SEM. Differential gene expression analyses to compare cells transfected with siRNA for *NOTCH1* or Scramble was performed using the limma package in R (v2.13.0). Calculated *P*-values from the moderate Student’s *t*-statistics were adjusted using the Benjamini–Hochberg method for multiple testing. Sample size and appropriate number of biological replicates has been discussed with our biostatistician for animals, imaging, and transcriptional/protein levels.

In multiple cases, collaborators performed the analysis of data in a manner that was blinded (cholesterol analysis, image analysis, atherosclerotic plaque burden).

### Data availability

The data set for the gene microarray analysis on HAECs treated with siNOTCH1 or scramble was deposited in the National Center for Biotechnology Information Gene Expression Omnibus database under accession number GSE72633. The rest of the data is available from the authors upon reasonable request.

## Electronic supplementary material

Supplementary Information

Description of Additional Supplementary Files

Supplementary Movie 1

Supplementary Movie 2
