# Supplementary material for: NOTCH1 is a mechanosensor in adult arteries
Source: Nat Commun. 2017 Nov 20;8:1620. doi: 10.1038/s41467-017-01741-8 (PMC5696341; doi:10.1038/s41467-017-01741-8)
Supplement: Supplementary file 1 — Supplementary Information [file 41467_2017_1741_MOESM1_ESM.pdf]

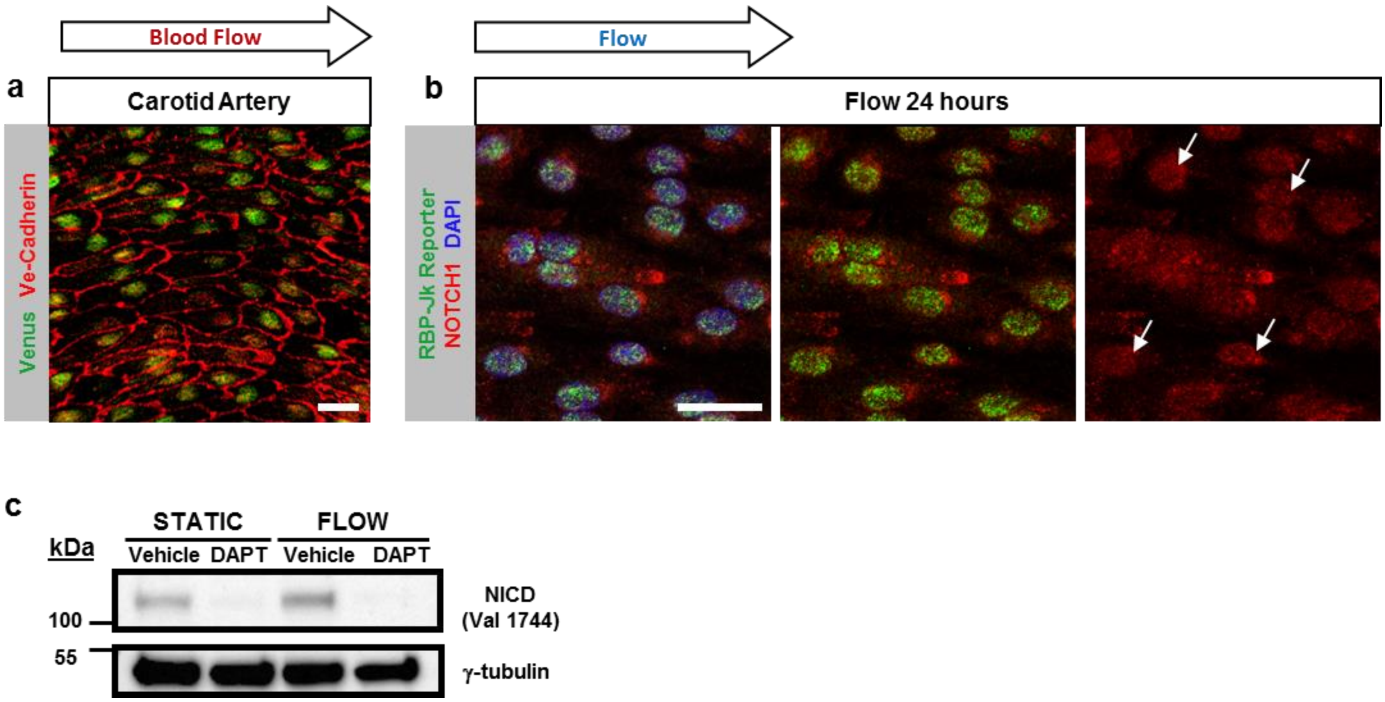

Supplementary Figure 1 Notch1 signaling is present *in vivo* in the endothelium of carotid artery and NOTCH1 activation is visualized *in vitro*. (a) *En face* imaging of carotid artery of the Venus Notch reporter mouse (RBP-Jk:H2B-Venus transgenic) to assess *Notch* signaling (n=5, scale bar=20µm). (b) HAECs transfected with RBP-Jk GFP reporter were subjected to flow (20 dynes cm^{-2}) for 24 hours and then fixed and stained for NOTCH1 and DAPI to confirm presence of NOTCH1 in the nucleus (white arrowheads, scale bar=20µm). (c) Immunoblot of protein lysates from endothelial monolayers cultured for 24 hours statically or in the presence of flow (20 dynes cm^{-2}) with DAPT or vehicle. NICD was used to identify cleaved Notch1 and γ-tubulin was used as loading control (BAECs, representative of n=4 biological replicates).

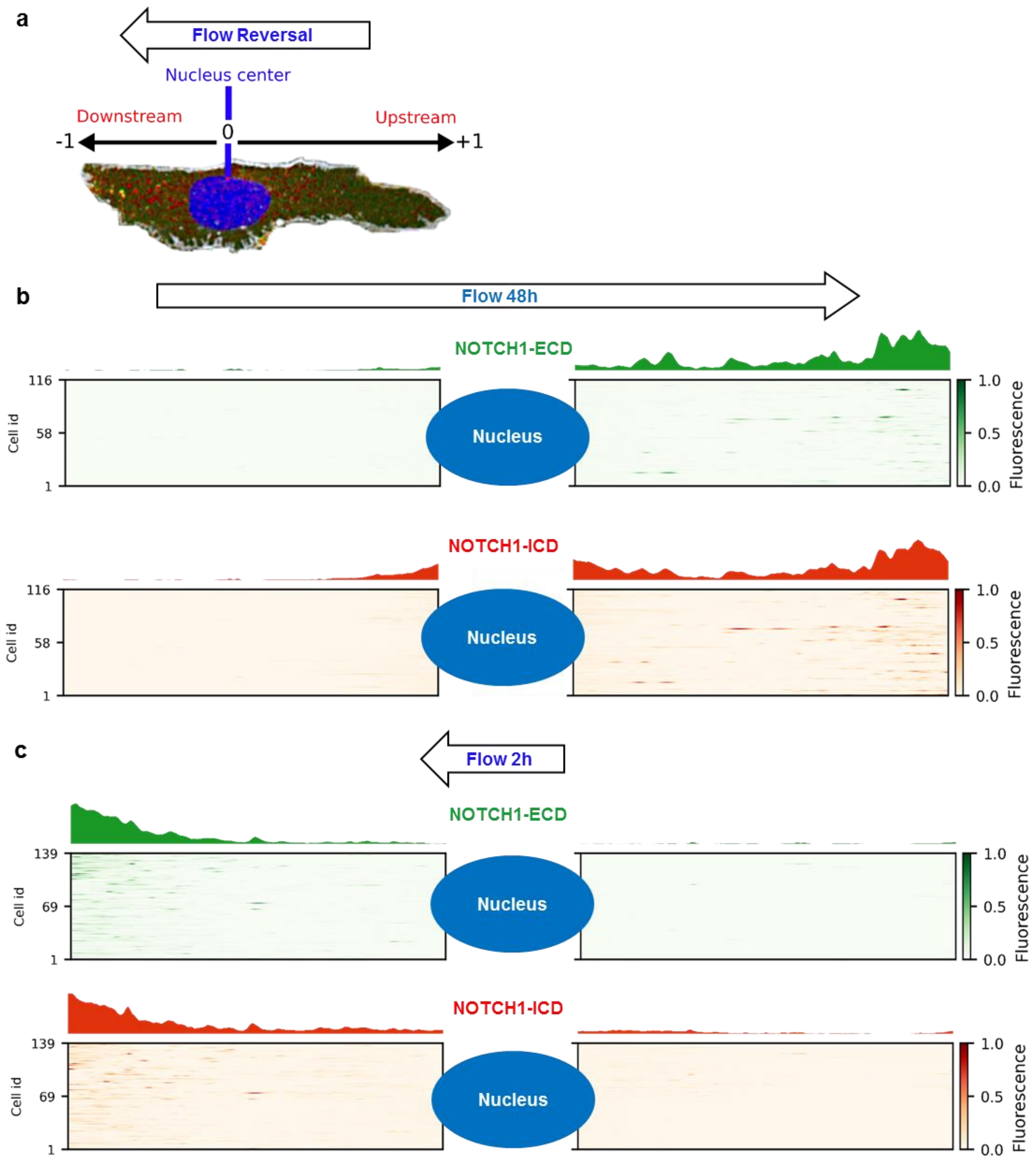

Supplementary Figure 2 Kinetics of NOTCH1 protein re-distribution upon flow direction reversal. (a) Endothelial cell dimensions were standardized using the nucleus as point of reference. The distance from each end to the center of the nucleus was normalized to 1. (b) The spatial distribution of NOTCH1 extracellular domain (ECD, green) and intracellular domain (ICD, red) along the flow axis after 48 hours of flow concentrates on the downstream pole (right-hand side). The raster plots present the spatial concentration per cell (bottom) with the average protein concentration plotted on top. Each line corresponds to an individual cell (n=116 cells evaluated). (c) After the application of reverse flow direction for 2 hours, the spatial distribution of both ECD and ICD is reversed along the flow axis to the new downstream pole (left-hand side) again plotting the spatial concentration per cell (bottom) and average protein concentration on top. Each line corresponds to an individual cell (n=139 cells evaluated).

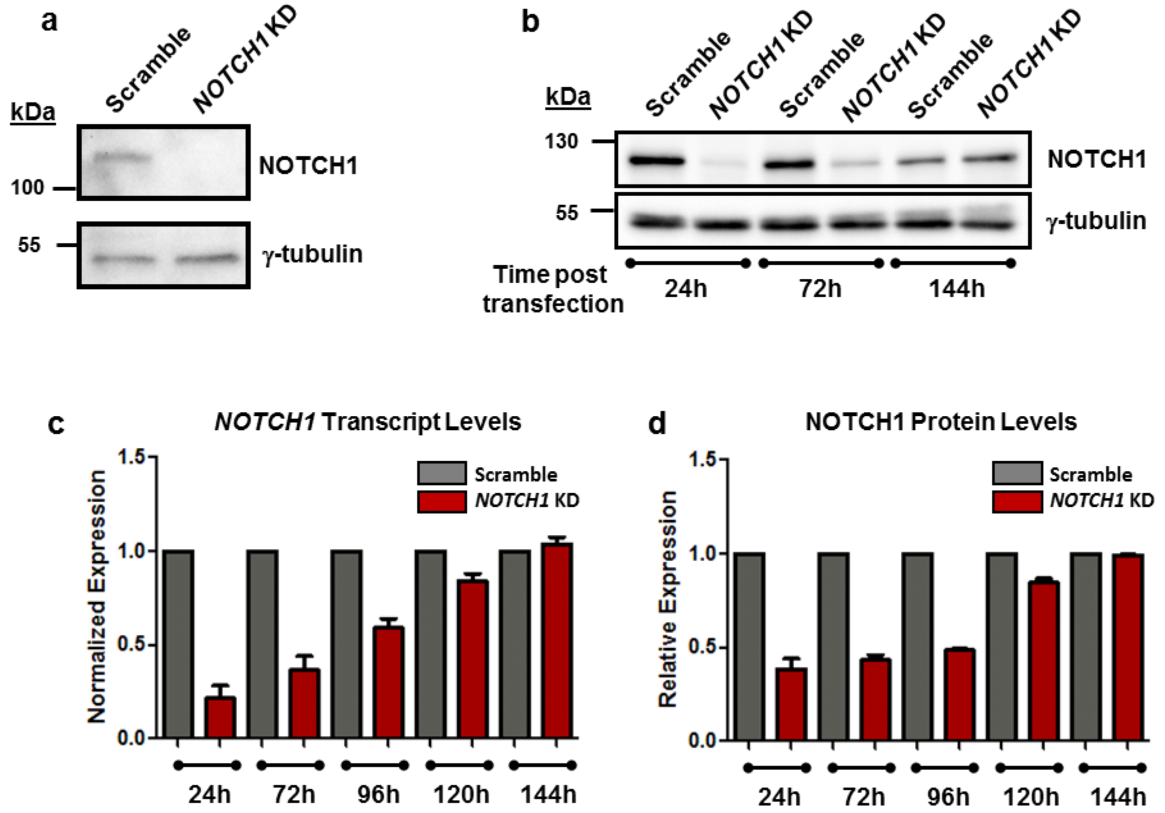

Supplementary Figure 3 Kinetics of siRNA mediated *NOTCH1* knock-down (KD) in HUVECs. (a) Representative immunoblot to confirm knock down of NOTCH1 protein at start of flow experiments, 24 hours post siRNA transfection. (b) Western blot of protein lysates from HUVECs harvested at specified time points post siRNA transfection: 24, 72 and 144 hours. Immunoblots were analyzed for NOTCH1 protein expression and normalized to γ-tubulin as loading control. (c) Quantification of *NOTCH1* transcript levels from 24 hours to 144 hours as measured by qPCR analysis. (d) Quantification of NOTCH1 protein expression levels from 24 hours to 144 hours post transfection for HUVECs cultured statically. Graph bars present mean ± SEM, n=5.

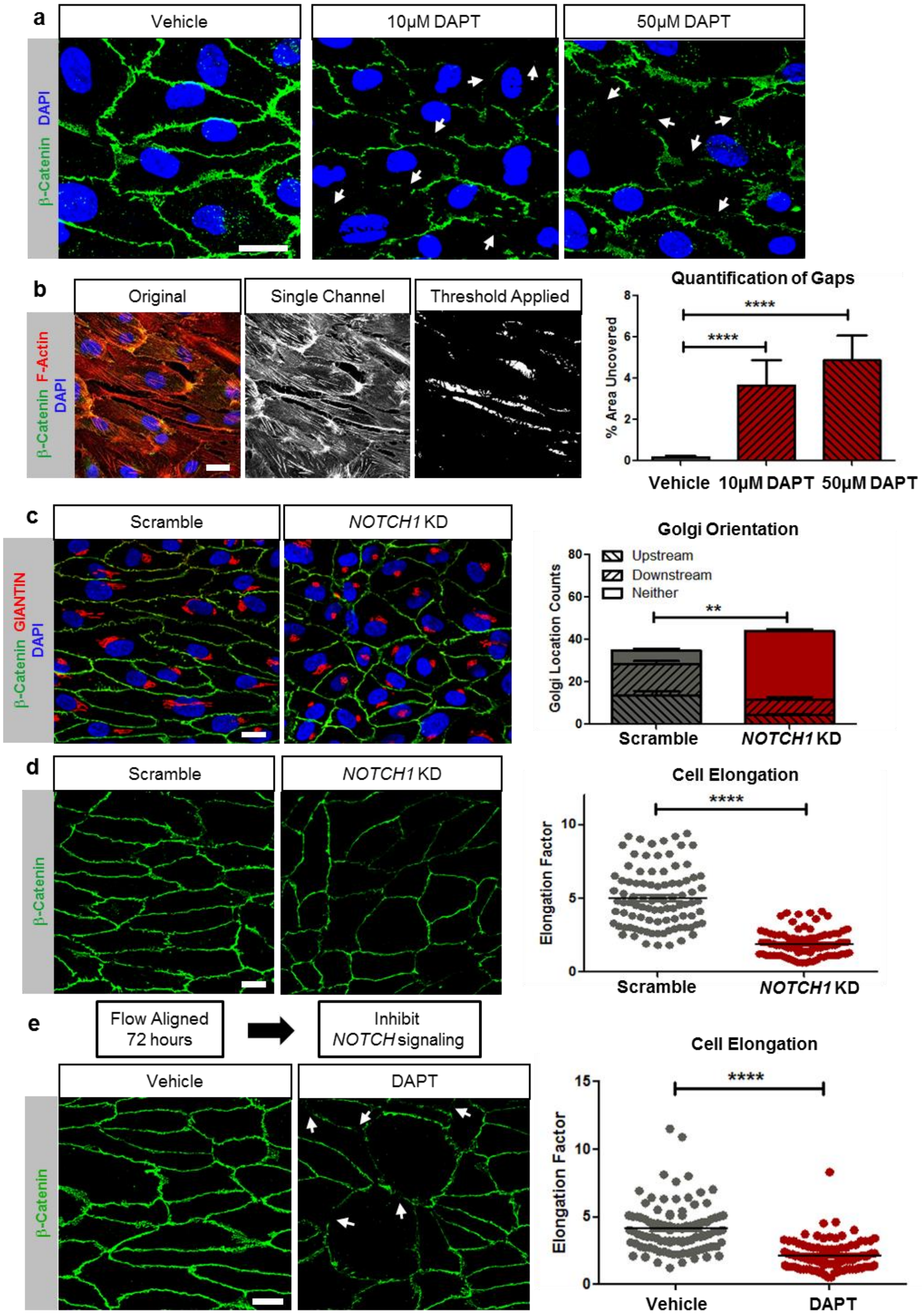

Supplementary Figure 4 Endothelial cell junctions and cell polarity under flow are compromised in the absence of NOTCH1 signaling. For all flow experiments, 10 dynes cm^{-2} was applied to HUVEC monolayers and 20 dynes cm^{-2} for HAEC monolayers to mimic physiological flow experienced *in vivo*. (a) HUVECs were cultured under flow for 24 hours in the presence of γ-secretase inhibitor DAPT or vehicle (volume equivalency of 50µM DAPT). DAPT treated monolayers displayed discontinuous junctions (indicated in white arrows) while vehicle treated monolayers showed no discontinuity. (b) Quantification of gaps for HUVEC monolayers subjected to flow for 72 hours with DAPT or vehicle. Gaps were quantified by applying a threshold on F-Actin channel and inverting the image to measure percent area uncovered. Graph shows mean ±SEM for 4 biological replicates. T test ****$P < 0.0001$. (c) Golgi location with respect to flow for HUVECs, siRNA for *NOTCH1* and Scramble. Quantification was performed on 4 biological replicates per condition. T test **$P < 0.01$. (d) HAECs treated with siRNA for *NOTCH1* or Scramble were flow-conditioned for 48 hours and quantified for cell elongation factor. Quantification of 120 cells from 4 biological replicates are plotted. Graph shows mean as horizontal bar. T test ****$P < 0.0001$. (e) HAECs were flow aligned for 72 hours and then treated with 50µM DAPT or vehicle for 24 hours under flow. Staining for cell-cell junctions revealed reduction in cell elongation for DAPT treated monolayers. Graph shows data obtained from 4 biological replicates with mean as horizontal bar. T test ****$P < 0.0001$. Scale bars=10µm.

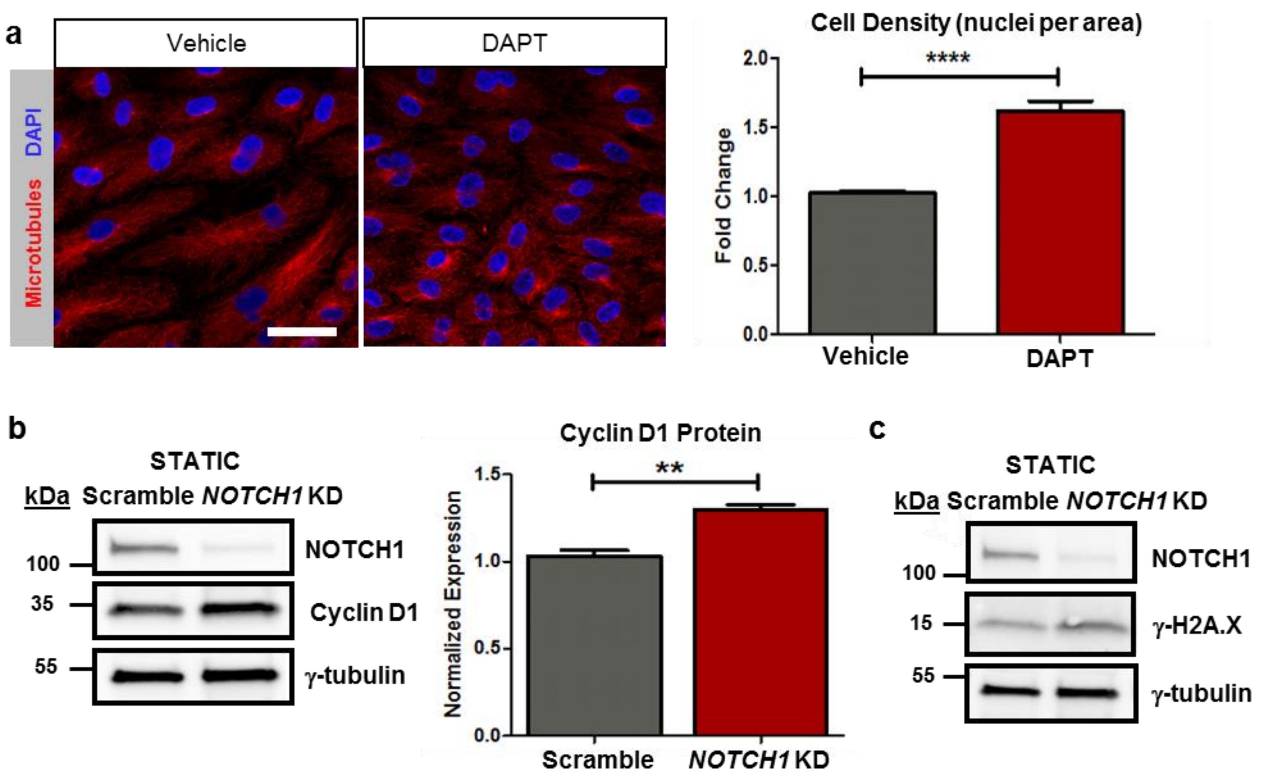

Supplementary Figure 5 Suppression of NOTCH1 signaling enhances endothelial cell proliferation. (a) Microtubule staining of endothelial monolayers subjected to shear stress in the presence of 50µM DAPT or vehicle (DMSO) for 72 hours. Cell nuclei were counted for both conditions to determine average cell density. Quantification for each condition displayed a significant increase in cell density for the DAPT treated cultures (HUVECs, 3 biological replicates, graph shows fold change in density after evaluation >140 cells per condition). Scale bar=20µm. T test $^{****}P < 0.0001$. (b) Immunoblot analysis of Cyclin D1 protein expression for statically cultured HAECs showed 1.3-fold enhancement for *NOTCH1* KD cells compared to control cells (n=3 biological replicates). T test $^{**}P < 0.01$. (c) Immunoblot for confluent static cultures (24 hours) of HAECs transfected with siRNA against *NOTCH1* or Scramble show an increase in γ-H2A.X protein levels for *NOTCH1* KD monolayers.

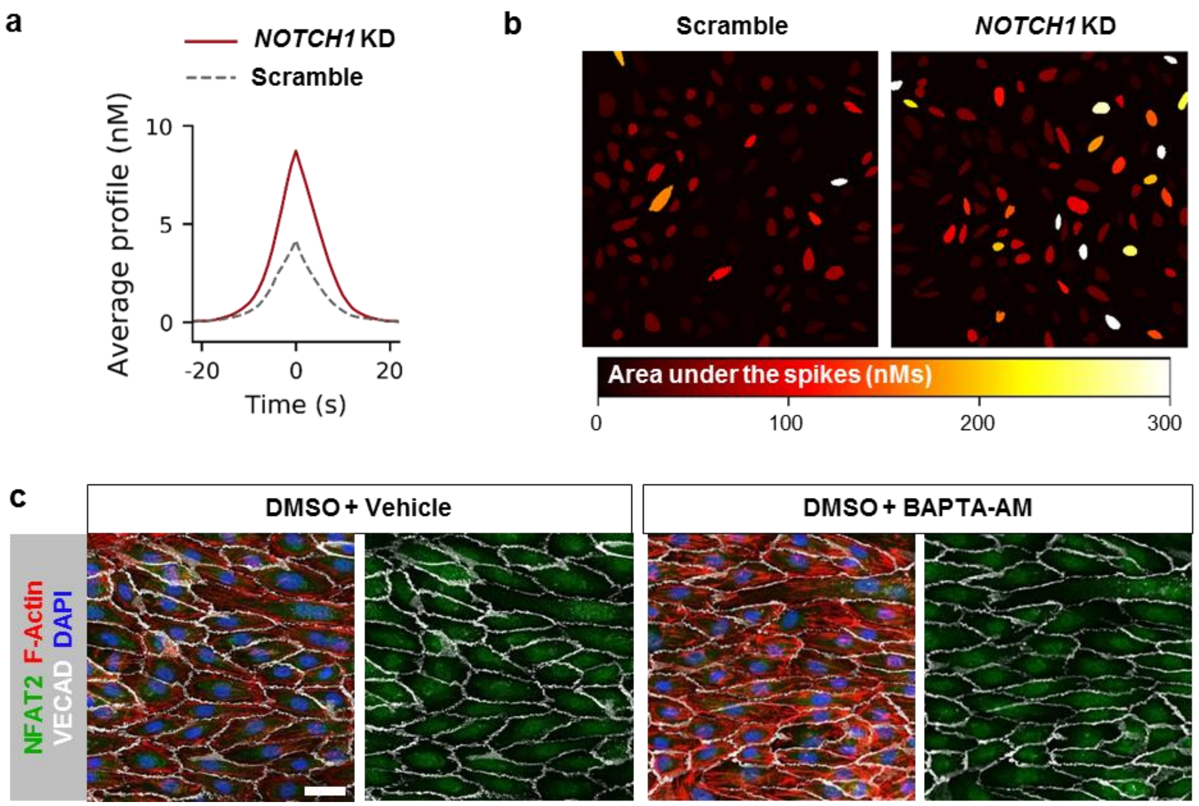

Supplementary Figure 6 Calcium dynamics in endothelial monolayers. (a) Average Ca^{2+} spike shape for *NOTCH1* KD (solid red line) and Scramble (grey dashed line) HAECs to show the average [Ca^{2+}] concentration for each: 3.9±0.3 nM for Scramble and 8.4±0.5 nM for *NOTCH1* KD. The average profile was obtained from measurements of more than 300 cells per group from 3 biological replicates. (b) Area under the spikes encoded for color overlaid on segmented cells from one replicate for Scramble and *NOTCH1* KD. (c) Control HAEC monolayers were treated with DMSO and BAPTA-AM or vehicle under flow to confirm that cell-cell junctions were maintained under control treatments. Scale bar=20µm.

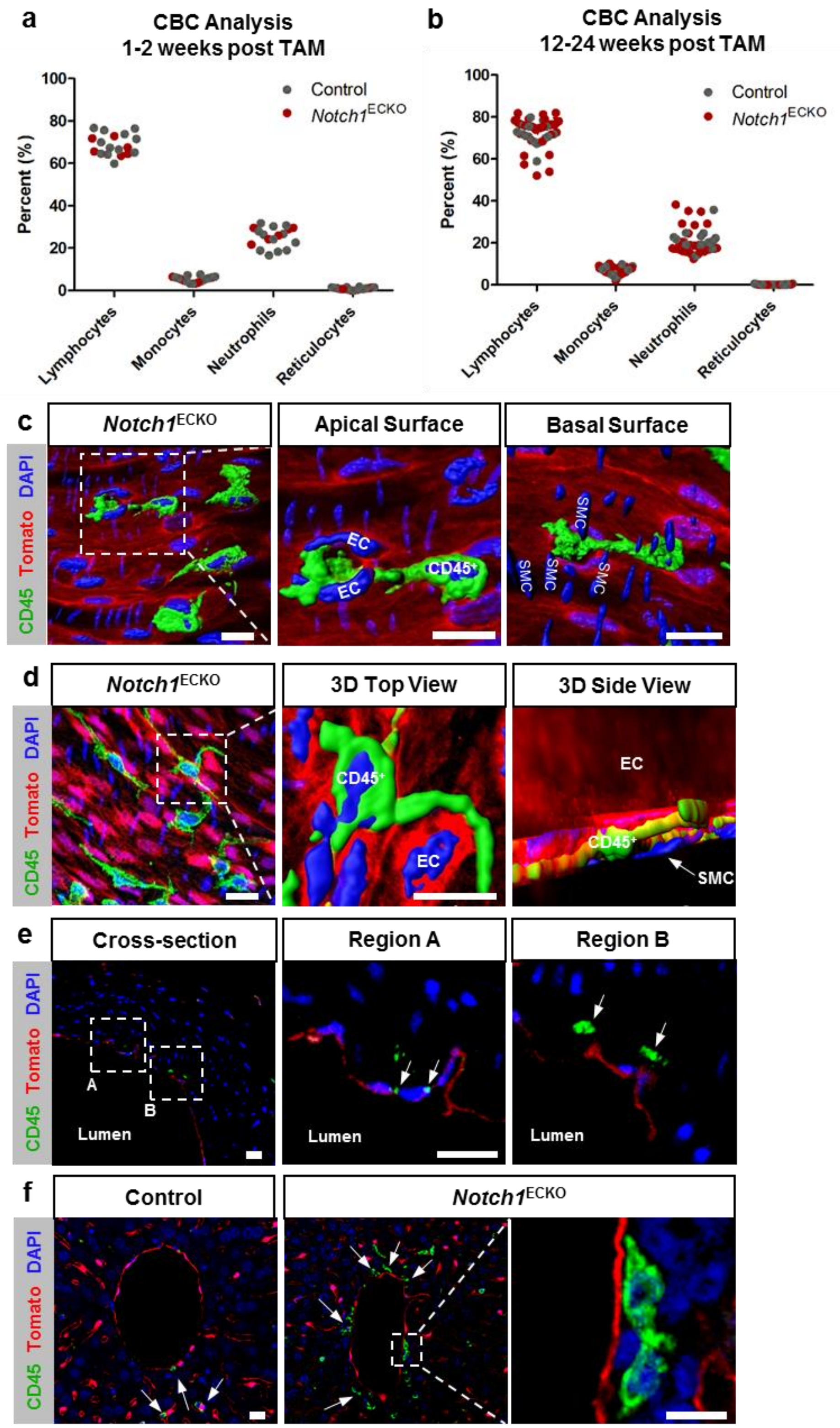

Supplementary Figure 7 Analysis of inflammatory phenotype for *Notch1*^{ECKO} animals. (a) Complete blood count (CBC) analysis of retro-orbital bleeds from control and *Notch1*^{ECKO} mice at 1 week and 2 weeks post tamoxifen injection. (b) CBC analysis of control and *Notch1*^{ECKO} mice at 12, 14, 16 and 24 weeks post tamoxifen. In both (a) and (b) the percent of each blood cell type is indicated in the graph (n=5-10 per group). (c) 3D reconstruction of *en face* confocal imaging to show the apical and basal surface of *Notch1*^{ECKO} aortic endothelium. (d) *En face* confocal imaging and 3D reconstruction to determine the topological location of inflammatory cells in the *Notch1*^{ECKO} aortic wall. 3D top view marks the green CD45^{+} cell and TdTomato labeled EC; rotating 45 degrees shows the same CD45^{+} cell sitting above the smooth muscle cell (SMC) layer. (e) Cross section of *Notch1*^{ECKO} aortic wall revealing CD45 staining at the endothelial layer and just below. (f) Histological cross sections of livers from control and *Notch1*^{ECKO} mice at 8 weeks post tamoxifen. Staining for CD45 shows an increase in inflammatory cells in the *Notch1*^{ECKO} liver. Scale bars=10µm.

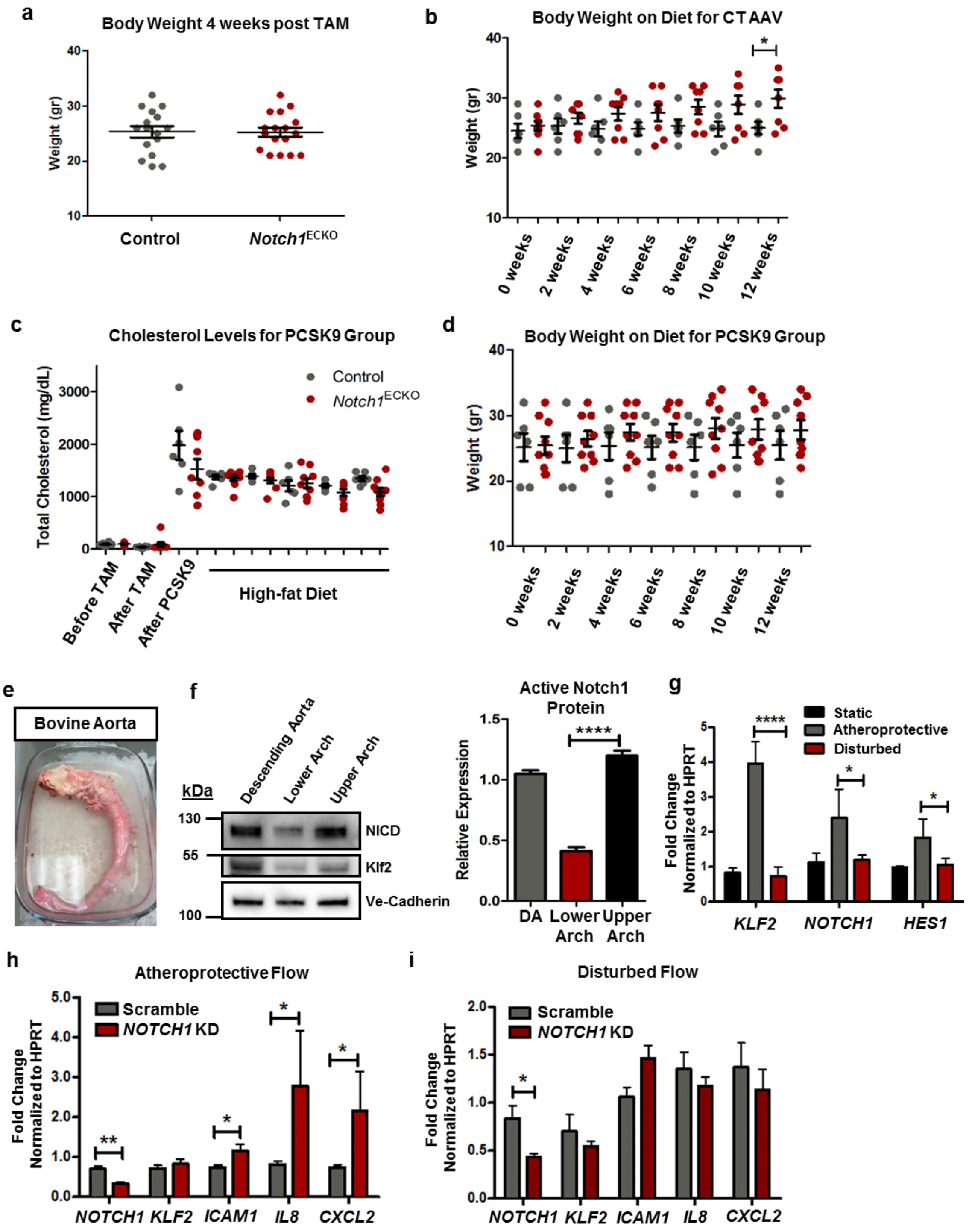

Supplementary Figure 8 Animal body weight and lipid analysis from hypercholesterolemia study and NOTCH1 signaling under different flow profiles. (a) Body weight for control and *Notch1*^{ECKO} animals 4 weeks post tamoxifen injection. Each dot represents one individual animal. Control mice are Cre^{+} tdTomato reporter^{+}. (b) Body weights for control and *Notch1*^{ECKO} animals injected with control AAV on a high-fat diet for 3 months (gray is control and red is *Notch1*^{ECKO}). (c) Cholesterol levels for PCSK9-AAV injected animals analyzed every two weeks. (d) Body weights for PCSK9-AAV injected animals on high-fat diet for 3 months. (e) Freshly isolated bovine aorta before dissection of arterial segments and protein lysis. (f) Immunoblot quantification of protein lysates from bovine endothelial arterial segments, quantification of NICD expression levels shown at right. Graph bars present mean ± SEM, evaluation of n=4 biological replicates. (g) Quantification of gene expression for HAECs subjected to 24 hours of static, atheroprotective or disturbed flow shows lack of up-regulation of *KLF2*, *NOTCH1* and *HES1* under disturbed flow. Graph bars represent mean ± SEM, n=4 biological replicates. T test ****$P<0.0001$, *$P<0.05$. (h) Under atheroprotective flow for 24 hours, *NOTCH1* KD HAECs show an up-regulation of inflammatory markers *ICAM1*, *IL8* and *CXCL2*. Graph bars represent mean ± SEM, n=6 biological replicates. Mann-Whitney *U* test, **$P<0.01$ and *$P<0.05$. (i) Under disturbed flow for 24 hours, inflammatory markers are not different between Scramble and *NOTCH1* KD HAECs. Graph bars represent mean ± SEM, n=3 biological replicates. T test *$P<0.05$.

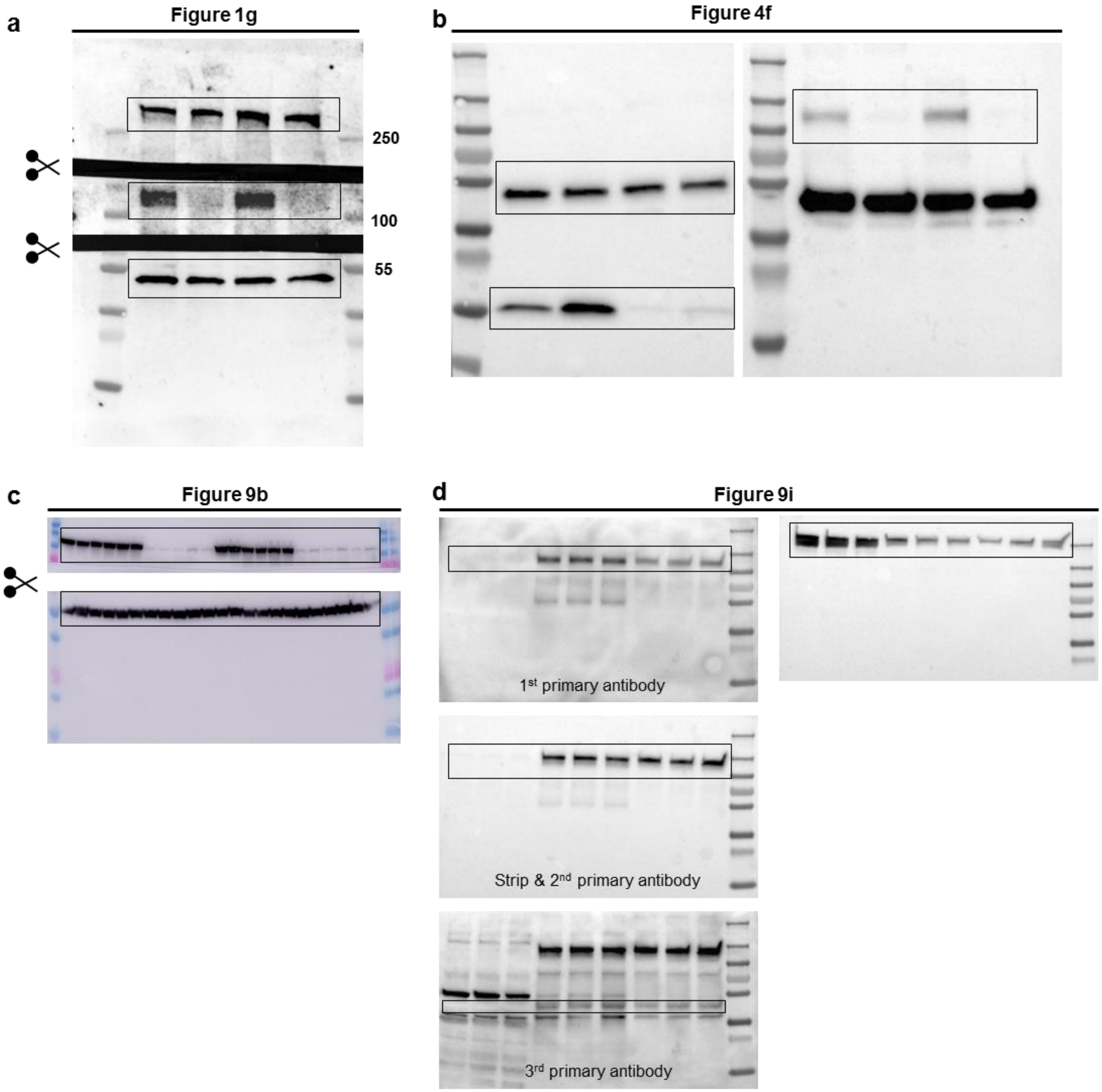

Supplementary Figure 9 Full view of the immunoblots used in main figures. The boxed regions indicate the bands shown in the figures.

Supplementary Table 1 Cell cycle genes changed by NOTCH1 reduction.

ILMN_ID	Gene Symbol	Gene Name	Fold Change	p-value	Function	Reference (PMID)
ILMN_1796216	VASH1	Vasohibin 1	4.28	1.80E-09	The expression of this gene inhibits migration, proliferation and network formation by endothelial cells as well as angiogenesis.	15467828
ILMN_3251550	PHLDA1	Pleckstrin Homology Like Domain Family A Member 1	4.19	1.80E-07	Also known as TDAG51. Transient overexpression of TDAG51 in HUVECs elicites significant changes in cell morphology, decreased cell adhesion, and promoted detachment-mediated programmed cell death	12738777
ILMN_1709683	RASSF2	Ras Association Domain Family Member 2	2.75	2.04E-07	RASSF2 has been shown to bind directly to K-Ras in a GTP-dependent manner via the Ras effector domain but not H-Ras. Moreover, RASSF2 functions as a tumor suppressor gene through its role promoting apoptosis and cell cycle arrest	12732644
ILMN_2219767	MYCN	V-Myc Avian Myelocytomatosis Viral Oncogene Neuroblastoma Derived Homolog	4.21	3.91E-07	Member of the myc family including MYC and MYCL. Functions to regulate a number of genes crucial for cell cycle control and differentiation in multiple pathways such as downregulating SKP2, TP53INP1 leading to decreased p21WAF1, downregulating of DKK1 upstream of the wnt/β-catenin pathway and modulation of apoptosis by upregulating of p53 and Mdm2.	20101214

ILMN_2376194	CAMK2B	CAMK2B calcium/calmodulin dependent protein kinase II beta	4.34	6.48E-07	The product of this gene encodes the beta subunit/isoform of the Ca2+/calmodulin-dependent protein kinase II (CAMKII). Dysregulation of CAMKII have been implicated as crucial in the progression of cancer trhough their regulation of cell proliferation and cell cycle.	25961153
ILMN_1688480	CCND1	Cyclin D1	2.29	8.29E-07	Cyclin D1 promotes cell proliferation as a regulatory partner for CDK4 or CDK6. Activation of cyclin D–CDK4/CDK6 initiates the release of the RB-dependent cell cycle-inhibition leading to progression through to S phase.	21734724
ILMN_2214197	TP53INP1	Tumor Protein P53 Inducible Nuclear Protein 1	1.97	1.42E-06	This protein is a stress-induced p53-target gene which in association with homeodomain-interacting protein kinase-2 (HIPK2), TP53INP1 phosphorylates p53 protein at Serine-46. This enhances p53 protein stability and its transcriptional activity, leading to transcriptional activation of p53-target genes such as p21 and PIG3, cell growth arrest and apoptosis upon DNA damage stress.	23717325
ILMN_1714383	TPD52L1	Tumor Protein D52-Like 1	2.58	1.55E-06	The encoded protein has been previously shown to interact with the mitogen-activated protein kinase kinase kinase 5 (MAP3K5/ASK1) and act to positively regulate MAP3K5-induced apoptosis.	14761963

ILMN_1707748	PIM3	Pim-3 Proto-Oncogene, Serine/Threonine Kinase	2.06	2.35E-06	The protein encoded by this gene belongs to the Ser/Thr protein kinase family, and PIM subfamily. PIM3 regulates phosphorylation of Bad at Ser112 and overexpression of PIM3 leads to increased proliferation in cells.	24789328
ILMN_1714730	UBE2C	Ubiquitin Conjugating Enzyme E2 C	1.93	1.95E-06	This protein is an ubiquitin-conjugating enzyme that plays a critical role with anaphase-promoting complex/cyclosome (APC/C) in progression of and exit from M phase. Temporal presence of the protein is regulated by autoubiquination and interaction with APC/C	10930472
ILMN_2337923	TPD52L1	Tumor Protein D52-Like 1	2.31	5.18E-06	The encoded protein has been previously shown to interact with the mitogen-activated protein kinase kinase kinase 5 (MAP3K5/ASK1) and act to positively regulate MAP3K5-induced apoptosis.	14761963
ILMN_1770085	BTG2	BTG Anti-Proliferation Factor 2	1.61	6.10E-06	The protein encoded by this gene is regulated by a p53-dependent mechanism and has been implicated in the regulation of cell cycle progression through G2/M phase, as loss of this protein does not impede cells with DNA damage progressing into M phase.	8944033
ILMN_1683927	ITGAE	Integrin Subunit Alpha E	2.09	2.48E-06	Also known as CD103. Interaction of CD103/Integrinβ7 with E-cadherin promotes the proliferation of	12414996

					CD8+ T-lymphocytes in vitro	
ILMN_1749829	DLGAP5	DLG Associated Protein 5	1.91	9.82E-06	Also known as HURP. HURP controls spindle stability and dynamics during mitosis through promoting microtubule polymerization and bipolar spindle formation, as well as decreasing the turnover rate of the mitotic spindle.	16769820
ILMN_1815154	MYH10	myosin heavy chain 10	2.52	3.63E-06	Also known as non-muscle myosin IIB (NMIIB). This myosin is required for meiotic cytokinesis in male mice and loss of this protein results in bi-nucleated secondary spermatocytes.	22820068
ILMN_1658494	C13orf15	Regulator of Cell Cycle	2.02	3.73E-06	Also known as RGCC and RGC32. This gene is thought to regulate cell cycle progression. It is induced by p53 in response to DNA damage, or by sublytic levels of complement system proteins that result in activation of the cell cycle. The encoded protein localizes to the cytoplasm during interphase and to centrosomes during mitosis. The protein forms a complex with polo-like kinase 1.	17146433
ILMN_2399523	JAG2	Jagged2	2.18	3.79E-06	A member of the Notch Receptor family of ligands. Ectopic expression of this protein increases cellular proliferation of B lymphoma cells in vitro.	20133585

ILMN_1789627	Sept5	Septin 5	2.29	1.60E-05	This gene is a member of the septin gene family of nucleotide binding proteins, appear to regulate cytoskeletal organization. Disruption of septins function disturbs cytokinesis and results in large multinucleate or polyploid cells.	22314400
ILMN_1766169	BCAT1	Branched Chain Amino Acid Transaminase 1	1.74	4.05E-06	This gene is a downstream target of the oncogene c-myc. Silencing of this gene inhibits cellular proliferation and G1/S transition.	18074675
ILMN_2181432	SPC24	SPC24, NDC80 Kinetochore Complex Component	1.87	8.32E-06	This protein serves as part of the Ndc80 complex that localizes at centrosomes during mitosis and functions in coordinating the process of chromosome segregation in mitosis.	17521635
ILMN_1663390	CDC20	Cell Division Cycle 20	2.02	9.99E-06	This protein interacts with The Anaphase Promoting Complex (APC) to regulate cell cycle progression. In particular, during metaphase, when all kinetochores are attached to microtubules, APC/CCdc20 ubiquitylates securin and cyclin B leading to the activation of the protease separase and inactivates the cyclin-dependent kinase-1 (Cdk1). Separase then cleaves cohesin complexes (shown as red circles) that are holding sister chromatids together and thereby initiates sister-chromatid	16896351

					separation and exit from mitosis by Cdk1 inactivation.	
ILMN_2349459	BIRC5	Baculoviral IAP Repeat Containing 5	1.80	1.57E-05	Also known as Survivin. This evolutionarily conserved protein plays a crucial role in mitosis through its roles in chromosomal attachment and spindle-assembly checkpoint.	18075512
ILMN_3239771	DLGAP5	DLG associated protein 5	1.96	1.59E-05	Also known as HURP. HURP controls spindle stability and dynamics during mitosis through promoting microtubule polymerization and bipolar spindle formation, as well as decreasing the turnover rate of the mitotic spindle.	16769820
ILMN_1766499	HSPA2		3.51	1.63E-05	Also known as HSP70-2. Functions in cell cycle. Loss of protein leads to impaired meosis in sperm due to impaired complex formation between paired homologous chromosomes during meiotic prophase.	9409676
ILMN_1705876	NAP1L1	Nucleosome Assembly Protein 1 Like 1	1.41	3.37E-04	Also known as NRP. This gene encodes a member of the nucleosome assembly protein (NAP) family. Studies in neuroendecrine tumor models suggest that this protein promotes cellular proliferation as an inhibitor of p57Kip2.	25071868

ILMN_1802669	PPP3CB	Serine/threonine-protein phosphatase 2B catalytic subunit beta isoform	1.60	2.32E-05	This gene encodes the beta isoform of the calmodulin-binding catalytic subunit of Calcineurin. In relation to cell cycle, Calcineurin functions through regulation of cyclin D and cdk4.	14671000
ILMN_1791726	TUBB3	Tubulin Beta 3 Class III	1.73	2.48E-05	β-tubulin isotype. Forms microtubules as heterodimer with α-tubulin. Due to the fact that elevated levels of this tubulin protein have been previously associated with poor cancer prognosis and in-vitro work showing that altered levels of this tubulin lead to drug resistance in both hematological and solid tumors, it has been strongly suggested this protein functions as a survival factor in cancer.	24995158
ILMN_1664434	TCF3	transcription factor 3	1.71	2.50E-05	Overexpression of this gene has been shown to negatively regulator cell proliferation and to promote apoptosis in classical Hodgkin lymphoma cell lines.	27166193
ILMN_1778242	CALM1	Calmodulin 1	1.72	3.66E-05	In vascular smooth muscle cells, calmodulin interacts with cyclin E1 to promote the progression from G1 to S phase.	16627785
ILMN_3187623	SYCE1L	synaptonemal complex central element protein 1 like	2.02	3.70E-05	Also known as mmrp2. Based on protein structure and mRNA expression levels this protein is strongly suspected to contribute to meiosis.	16328886
ILMN_1741459	CDK10	Cyclin Dependent Kinase 10	1.55	1.14E-03	Also known as PISSLRE. This kinase has been shown to play a role in cellular	7664269

					proliferation and dominant negative versions of this protein arrest cells in the G2/M phase of the cell cycle.	
ILMN_1684439	MLF1	Myeloid Leukemia Factor 1	2.36	4.73E-05	The protein MLF1 functions as a negative regulator of cell cycle progression upstream of the tumor suppressor p53 by regulating the endogenous level of COP1, a ubiquitin ligase for p53	15861129
ILMN_1773119	CCNF	Cyclin F	1.71	4.91E-05	Cyclin F is the founding member of the F-box family of proteins, which are the substrate recognition subunits of Skp1-Cul1-F-box protein (SCF) ubiquitin ligase complexes. SCF Cyclin F is involved in relation to cell cycle is involved in centrosome duplication, and spindle formation.	23182110
ILMN_1718565	CDKN1C	Cyclin Dependent Kinase Inhibitor 1C	1.56	1.29E-02	Also known as P57/Kip2. The encoded protein functions as a potent inhibitor of G1- and S-phase CDKs including cyclin E-cdk2, cyclin D2-cdk4, and cyclin A-cdk2 and, to lesser extent, of the mitotic cyclin B-Cdc2.	7729683

Supplementary Table 2 Inflammatory genes changed by NOTCH1 reduction.

Probe ID	Gene Symbol	Full Name	Fold Change	p-value	Function	Reference (PMID)
ILMN_1720048	CCL2	C-C Motif Chemokine Ligand 2	2.14	3.06E-07	Also known as MCP-1. Functions as a potent chemoattractant for monocytes in vitro and induces the expression of integrins required for chemotaxis in monocytes	1348518
ILMN_1739393	SELE	Selectin E	3.22	1.11E-06	Also known as E-selectin. E-selectin is expressed by cytokine-stimulated endothelial cells and is thought to be responsible for the accumulation of blood leukocytes at sites of inflammation by mediating the adhesion of cells to the vascular lining.	12829015
ILMN_2365307	CD276	CD276 Molecule	2.24	1.24E-06	Also known as B7H3. Expression is induced on dendritic cells (DCs) and monocytes by inflammatory cytokines and this molecule functions to enhance the induction of cytotoxic T cells while selectively stimulating interferon gamma (IFN-gamma) production in the presence of T cell receptor signaling.	11224528
ILMN_1801584	CXCR4	C-X-C Motif Chemokine Receptor 4	2.95	1.67E-06	Also known as LESTER. Expressed on neutrophils, myeloid cells, and T lymphocytes, functions as an essential co-factor for HIV type 1 and type 2 entry in to CD4+ cells. Primary receptor	10807766

					for the CXC chemokine SDF-1, mice lacking the CXCR4 gene exhibit impaired B lymphopoiesis, myelopoiesis, hematopoiesis, derailed cerebellar neuron migration, and defective formation of large vessels supplying the gastrointestinal tract.	
ILMN_1753823	IL17D	Interleukin 17D	1.84	5.89E-06	IL17D treatment of endothelial cells has been shown to stimulate the production of other cytokines including IL6, IL8 and GM-CSF. The increased expression of IL8 induced by this cytokine was found to be NF-kappa B-dependent.	12097364
ILMN_1682636	CXCL2	C-X-C Motif Chemokine Ligand 2	1.99	8.42E-06	Also known as GRO-β/MIP2. Acts a neutrophil and basophil leukocyte attractant and hematoregulatory chemokine whose effects depend on the maturity of the hemopoetic progenitors being treated.	9242519
ILMN_2320888	CXCR4	C-X-C Motif Chemokine Receptor 4	2.94	1.13E-05	Also known as LESTER. Expressed on neutrophils, myeloid cells, and T lymphocytes, functions as an essential co-factor for HIV type 1 and type 2 entry in to CD4+ cells. Primary receptor for the CXC chemokine SDF-1, mice lacking the CXCR4 gene exhibit impaired B lymphopoiesis, myelopoiesis, hematopoiesis,	10807766

					derailed cerebellar neuron migration, and defective formation of large vessels supplying the gastrointestinal tract.	
ILMN_1791447	CXCL12	C-X-C Motif Chemokine Ligand 12	2.50	1.39E-05	Also known as stromal derived factor 1 (SDF1). Chemoattractant that acts on T-lymphocytes and B-cells through interactions with its receptor CXCR4. Can also interact with the C-X-C chemokine receptor CXCR7.	24966838
ILMN_1740609	CCL15	C-C Motif Chemokine Ligand 15	1.70	1.94E-05	Chemotactic factor that attracts T-cells and monocytes, but not neutrophils, eosinophils, or B-cells. Acts mainly via CC chemokine receptor CCR1.	9624581
ILMN_1746175	TNFSF4	Tumor Necrosis Factor Superfamily Member 4	1.60	3.30E-05	Also known as Glycoprotein Gp34 and OX40 Ligand. TNFSF4 functions in T cell antigen-presenting cell (APC) interactions and mediates adhesion of activated T cells to endothelial cells.	8642328
ILMN_1689088	COLEC12	Collectin Subfamily Member 12	1.90	3.79E-05	Also known as CL-P1. Acts as a scavenger receptor that can bind and phagocytose bacteria (Escherichia coli and Staphylococcus aureus) and yeast (Saccharomyces cerevisiae). Furthermore, it reacts with oxidized low density lipoprotein (OxLDL), suggesting a role in atherogenesis	11564734

ILMN_1759787	THBD	Thrombomodulin	2.49	4.18E-05	Thrombomodulin is an endothelial-specific type I membrane receptor that binds thrombin. This binding results in the activation of protein C, leading to the degradation of clotting factors Va and VIIIa.	21805323
ILMN_1689111	CXCL12	C-X-C Motif Chemokine Ligand 12	2.22	7.02E-05	Also known as stromal derived factor 1 (SDF1). Chemoattractant that acts on T-lymphocytes and B-cells through interactions with its receptor CXCR4. Can also interact with the C-X-C chemokine receptor CXCR7.	24966838
ILMN_1787897	CXCL1	C-X-C Motif Chemokine Ligand 1	2.32	1.78E-04	Also known as GRO-α. It functions as a neutrophil-specific chemoattractant that is secreted by activated mononuclear cells along with IL-8.	9242519

Supplementary Table 3 Regulators of intracellular calcium genes changed by NOTCH1 reduction.

ILMN_ID	Gene Symbol	Gene Name	Fold Change	p-value	Function	Reference (PMID)
ILMN_1684795	PRND	prion like protein doppel	16.16	3.60E-10	PrPc limits the agonist-stimulated ER Ca2+ release and Ca2+ accumulation by mitochondria.	15788568
ILMN_2361768	CHRNA1	cholinergic receptor nicotinic alpha 1 subunit	6.12	1.72E-09	Encodes the alpha subunit of the acetylcholine receptor. In the embryo, stimulation with acetylcholine drives muscle contraction through intracellular calcium release.	16249237
ILMN_1796216	VASH1	vasohibin 1	4.28	1.80E-09	This protein is a downstream target of the calcium sensitive S100A4.	
ILMN_1678403	TMEM178	transmembrane protein 178	5.96	2.57E-09	Tmem178 localizes to the ER membrane and modulates RANKL-induced calcium fluxes to regulate NFAT1c activity in relation to bone mass	26644563
ILMN_1800225	PPARG	peroxisome proliferator activated receptor gamma	4.29	1.59E-08	Cardiomyocytes from mice overexpressing PPARg show abnormal calcium handling characterized by an increase amplitude in calcium transits and increased cardiac store in the sarcoplasmic reticulum	27154230
ILMN_1750800	ACO1	aconitase 1	3.38	2.36E-08	This gene encodes the TCA enzyme aconitase. Disrupition of aconitase activity by Fluoroacetate disrupts calcium signaling in glial cells leading to impaired uptake by connexin-43 hemichannels.	27784805

ILMN_1689353	APLN	apelin	3.20	6.79E-08	Apelin protects sarcoplasmic reticulum function and cardiac performance in ischaemia-reperfusion by attenuating oxidation of sarcoplasmic reticulum Ca2+-ATPase and ryanodine receptor.	23771946
ILMN_1655077	PRDM1	PR domain containing 1, with ZNF domain	6.41	2.25E-07	Also known as Blimp1. Is a known target of RANKL-NFAT1c signaling during osteoclastogenesis leading to decreased bone density	20133620
ILMN_2153916	HSPA2	heat shock protein 2	3.10	2.84E-07	Also known as HSP70-2. Functions in cell cycle. Loss of protein leads to impaired meosis in sperm. Shown to be calcium-regulated protein that resides on the plasma membrane of human sperm.	20078857
ILMN_1671106	GJA4	gap junction protein alpha 4	3.76	3.72E-07	Also known as connexin-37. This protein contributes to nitrogen oxide mediated calcium signal propagation between endothelial and smooth muscle cells at myoendothelial junctions	24885166
ILMN_1779071	FEZ1	fasciculation and elongation protein zeta 1 (zygin I)	2.15	4.66E-07	This gene encodes the homologue of C. elegans protein UNC-76. This protein has been shown to be involved in numerous cellular processes such as microtubule organization, neuronal differentiation and cargo transport. Known interaction	11856312

					partners include near BRCA1 (NRB1) and Calcium and Integrin Binding Protien (CIB).	
ILMN_1688780	S100A4	S100 calcium binding protein A4	2.89	5.16E-07	This gene encodes a small calcium protiein that is frequently overexpressed in lung cancer and promotes cell growth and motility.	24732359
ILMN_2376194	CAMK2B	calcium/calmodulin-dependent protein kinase II, beta	4.34	6.48E-07	The product of this gene encodes the beta subunit/isoform of the Ca2+/calmodulin-dependent protein kinase II (CAMKII). Dysregulation of CAMKII have been implicated as crucial in the progression of cancer through their regulation of cell proliferation and cell cycle.	25961153
ILMN_2059535	PPM1F	protein phosphatase 1F (PP2C domain containing)	2.43	1.32E-05	Also known as CaMKP. Interacts with neurofilament L (NFL) and prevents it from filament association.	27369073
