# Supplementary material for: NOTCH1 is a mechanosensor in adult arteries
Source: Nat Commun. 2017 Nov 20;8:1620. doi: 10.1038/s41467-017-01741-8 (PMC5696341; doi:10.1038/s41467-017-01741-8)
Supplement: Supplementary file 2 — Description of Additional Supplementary Files [file 41467_2017_1741_MOESM2_ESM.pdf]

Description of Additional Supplementary Files

File Name: Supplementary Movie 1
Description: **Calcium imaging of control (siScramble) endothelial cells.** Left: A confluent monolayer of control HAECs loaded with fluorescent Ca^{2+} indicator Oregon Green BAPTA-1 were recorded for changes in fluorescence intensity under laminar flow for 10 minutes. The first 25 seconds were removed due to camera saturation and then compressed to 20 seconds with a frame rate of 30 frames per second, scale bar=20μm. To remove photobleaching, the fluorescence was corrected with a linear decay model fitted to the averaged pixel intensity. Right: Fluorescence time series of a single cell (marked by red square on left panel). The raw time series without photobleaching correction is displayed in the top panel and the bottom panel plots the calcium concentration calculated by correcting the photobleaching and using F_{max}/F_{min}.

File Name: Supplementary Movie 2
Description: **Calcium imaging of *NOTCH1* KD (si*NOTCH1*) endothelial cells.** Left: A confluent monolayer of *NOTCH1* KD HAECs loaded with fluorescent Ca^{2+} indicator Oregon Green BAPTA-1 were recorded for changes in fluorescence intensity under laminar flow for 10 minutes. The first 25 seconds were removed due to camera saturation and then compressed to 20 seconds with a frame rate of 30 frames per second, scale bar=20μm. To remove photobleaching, the fluorescence was corrected with a linear decay model fitted to the averaged pixel intensity. Right: Fluorescence time series of a single cell (marked by red square on left panel). The raw time series without photobleaching correction is displayed in the top panel. In the bottom panel, the calcium concentration is plotted by correcting the photobleaching and using F_{max}/F_{min}.
